# Yeast Glucan Remodeling Protein Bgl2p: Amyloid Properties and the Mode of Attachment in Cell Wall

**DOI:** 10.3390/ijms252413703

**Published:** 2024-12-22

**Authors:** Nikita A. Motorin, Gennady I. Makarov, Valentina V. Rekstina, Evgeniy G. Evtushenko, Fanis A. Sabirzyanov, Rustam H. Ziganshin, Alexey K. Shaytan, Tatyana S. Kalebina

**Affiliations:** 1 Faculty of Biology, Lomonosov Moscow State University, Moscow 119991, Russia; n.motorin@intbio.org (N.A.M.); rekstinavv@my.msu.ru (V.V.R.); biophoenix@mail.ru (F.A.S.); shaytan_ak@mail.bio.msu.ru (A.K.S.); 2 Laboratory of the Multiscale Modeling of Multicomponent Materials, South Ural State University, Chelyabinsk 454080, Russia; makarovgi@susu.ru; 3 Faculty of Chemistry, Lomonosov Moscow State University, Moscow 119991, Russia; evtushenko@enzyme.chem.msu.ru; 4 Shemyakin and Ovchinnikov Institute of Bioorganic Chemistry, Russian Academy of Sciences, Moscow 117997, Russia; rustam.ziganshin@gmail.com

**Keywords:** cell wall, glucan remodeling proteins, glucanosyltransglycosylases, Bgl2p, AlphaFold, amyloid proteins, mode of attachment

## Abstract

Bgl2p is a major, conservative, constitutive glucanosyltransglycosylase of the yeast cell wall (CW) with amyloid amino acid sequences, strongly non-covalently anchored in CW, but is able to leave it. In the environment, Bgl2p can form fibrils and/or participate in biofilm formation. Despite a long study, the question of how Bgl2p is anchored in CW remains unclear. Earlier, it was demonstrated that Bgl2p lost the ability to attach in CW and to fibrillate after the deletion of nine amino acids in its C-terminal region (CTR). Here, we demonstrated that a Bgl2p anchoring is weakened by substitution Glu-233/Ala in the active center. Using AlphaFold and molecular modeling approach, we demonstrated the role of CTR on Bgl2p attachment and supposed the conformational possibilities determined by the presence or absence of an intramolecular disulfide bond, forming by Cys-310, leading to accessibility of amyloid sequence and β-turns localized in CTR of Bgl2p for protein interactions. We hypothesized the mode of Bgl2p attachment in CW. Using atomic force microscopy, we investigated fibrillar structures formed by peptide V187MANAFSYWQ196 and suggested that it can serve as a factor leading to the induction of amyloid formation during interaction of Bgl2p with other proteins and is of medical interest being located close to the surface of the molecule.

## 1. Introduction

Bioinformatic analysis of the yeast proteome revealed a high representation of β-aggregation-prone proteins in the cell wall (CW) [1]. Many of them play an important role in the development of candidiasis [2,3,4]. One of the widely investigated CW proteins with amyloid properties is a conservative, major, non-covalently attached protein, Bgl2p [5,6,7,8,9,10,11,12].

Bgl2p is present in CW of many species of ascomycete fungi and yeasts, such as *Aspergillus fumigatus* and *Saccharomyces cerevisiae*, and some species of pathogenic yeasts, such as *Candida albicans* [13,14,15,16] and demonstrates a high degree of homology [5,8,9,16]. This protein belongs to the glycosyl hydrolases of the GH17 family [17]. It plays an important role as one of the main glucan remodeling enzymes of CW and also protects yeast cells from drying and heating [18,19]. Bgl2p is significant in the formation of biofilm under sherry fermentation [20]. It was demonstrated that Bgl2p plays an important role in the virulence of pathogenic yeast species and has an adhesin-like role [3,8,9]. From the medical perspective, Bgl2p is an important antigen; the production of anti-Bgl2p antibodies in patients during the development of systemic candidiasis may serve as a positive prognosis for the disease progression [21].

In more than 30 years of studying this protein, there have been a lot of experiments demonstrating possible amyloid properties of Bgl2p in CW. Being incorporated in CW, it is resistant to treatment with 1% SDS at 37 °C for 1 h [10,11]. It was shown that in Bgl2p-containing CW preparations partially hydrolyzed by glucanase when stained with amyloid-specific Congo Red dye, zones with green birefringence are formed, while such zones were not detected in the CW preparation of the *bgl2Δ* strain [10].

The mode of attachment of this protein in CW is still unclear. Bgl2p is strongly attached in CW. However, covalent bonds are not involved in CW attachment of this protein [10,11]. Strong amyloidogenic amino acid sequences F_83_TIFVGV_89_, N_190_AFS_193_ and G_268_VNVIVFEA_276_ were predicted in the primary structure of Bgl2p by six computational algorithms FoldAmyloid, AGGRESCAN, DHPRED, Waltz, PASTA, TANGO [11]. One may think it is possible that amyloidogenic amino acid sequences within the molecule of the protein play an important role in the formation of amyloid-like structures and are responsible for its attachment. However, Bgl2p can be extracted from CW under heating (70–100 °C), even in water, which is not specific for amyloid proteins. The above makes it possible to suppose a potential ability of Bgl2p to form unusual, labile-to-heating amyloid-like structures. Nevertheless, after extraction under heating, Bgl2p forms fibrils detected with antibodies in the incubation medium [11]. Experiments using Thioflavin T (ThT) and Congo Red dyes and circular dichroism spectrum investigation demonstrated the proneness of this protein to β-aggregation after isolation from CW using heating [10,11,22,23]. This protein can be almost completely extracted from CW by heating to 100 °C in Laemmli buffer [10]. It should be noted that heating is an efficient way of quantitative extraction of Bgl2p from CW, but part of its molecules can be extracted by Tris under alkaline conditions, while the rest of the protein continues to be attached in CW and can be extracted by incubation with guanidine hydrochloride (GHChl). After that, Bgl2p forms structures morphologically resembling fibrils of glucagon [24]. Bgl2p molecules from Tris and GHChl fractions demonstrated differences in their posttranslational modifications [25].

Despite the above, when yeast is growing in presence of alcohol, for example under sherry fermentation conditions [20] or during cultivation of *S. cerevisiae ssu21/mcd4* strain, where the pathway of the GPI anchor biosynthesis is disrupted whereby CW lacks GPI-anchor proteins [26] and Bgl2p leaves CW [11,20,26] and can be detected in the culture medium in fibrillar form, but different than after heating [11]. These facts indicate that the structure of a Bgl2p molecule provides for conformational changes that allow it both to be strongly attached in CW and to leave CW for the environment in vivo under physiological conditions.

An important fact for understanding the unusual Bgl2p CW incorporation is the loss of this protein’s ability to attach in CW after the deletion of nine amino acids in the C-terminal region (CTR) of its molecule. That region includes a potentially amyloidogenic sequence that was identified as having amyloidogenic properties using only three algorithms [11], also one of four cysteine residues of this protein—Cys-310 and left-handed polyproline-II helices according to SwissModel [19]. A protein lacking CTR was identified in the culture medium, but it is unable to form fibrils in culture fluid [19]. It remains unclear whether this protein can form fibrils with amyloid structure, be incorporated into CW or after extraction, or possibly fibrillate in both cases but in a different manner.

In addition to the data already listed, it is important to note that Bgl2p has no carbohydrate-binding module but demonstrates glucan-binding properties [5].

It can be concluded that experimental data allowing us to understand the mode of Bgl2p attachment in CW are currently insufficient. In this work, we made use of computer modeling and experimental efforts to better understand the issue.

In recent years, the AlphaFold algorithm has become available, which outperforms other algorithms in terms of the quality of protein structure prediction [27,28]. Here, we have compared Bgl2p structural model predictions using various in silico prediction algorithms (I-Tasser, Robetta, SwissModel and AlphaFold). We compared Bgl2p models by analyzing the potential energies of globules and entropies of torsion angles, which describe conformations of peptide chains. We found that the AlphaFold model is characterized by the lowest entropy and potential energy among all the considered Bgl2p models, so this model was chosen for further consideration. Using this model, we performed extensive molecular dynamics simulations of Bgl2p and revealed that, in contrast to SwissModel, the C-terminal region of the protein in the AlphaFold model contains disulfide bond Cys-262–Cys-310 and an unstructured region capable of β-turn conformation. The result of molecular dynamics simulations of mutant form Bgl2pΔC_305–313_ suggested that the C-terminal region may contribute to its amyloidogenic properties and, along with β-turns, provide Bgl2p attachment to CW.

In an additional effort using molecular modeling and an experimental approach, we investigated the ability of Bgl2p with a disrupted active center to anchor in the cell wall. We showed that fibrillar structures can be formed by a peptide V_187_MANAFSYWQ_196_ with amyloid properties. We critically analyzed the obtained experimental and modeling data in the context of previously known results and proposed in this work a hypothesis for the attachment of the studied protein in the cell wall and its ability to form fibrils. The ability of the studied protein, whose molecule includes amyloid amino acid sequences, to form fibrils outside the cell is important for medicine.

## 2. Results and Discussion

### 2.1. Prediction and Choosing of Bgl2p Structural Model for Further Analysis

The crystallographic structure of Bgl2p has not been solved yet. To build a structural model of this protein, we applied molecular modeling using the most appropriate approach.

There are many tools for predicting the spatial structure of a protein by homology or by machine learning, some of which are presented in the Methods. It was necessary to propose a way to compare the quality of models obtained by these methods, which would allow us to compare models built by different tools. An obvious and natural way would be to calculate the potential energy of a protein globule for different models using the same tool, for example, the GROMACS package. However, it is also essential to consider the ordering of the protein globule, as it may prove to be, that a molten or partially unfolded molecule will have a lower potential energy compared to a folded molecule upon molecular dynamics simulations, which may not be advantageous in terms of the entropy (for example, due to the exposure of the hydrophobic core of the globule). The predicted model should exhibit an ordered arrangement of the peptide backbone. As a measure of order, it would be appropriate to use an estimation of the entropy associated with the torsion angles describing the conformation of the peptide chain. For this purpose, we decided to employ the entropy estimation method based on the compressibility of the discrete representation [29] and take into account both the estimates of the entropy of torsion angles and the potential energy of a protein globule excluding ions and water from consideration. This approach enables one [29] to identify the conformations of the peptide chain, which are observed in the data of X-ray diffraction analysis from conformations obtained by molecular dynamic simulations. These conformations are characterized by both low entropy and low potential energy.

**Figure 1:**
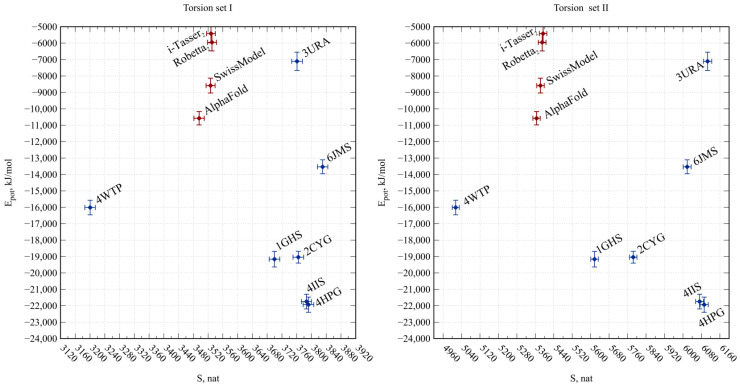
Potential energies of globules and entropies of its torsion angles for models of Bgl2p from *Saccharomyces cerevisiae* (red) and homologous glycosidases from the GH17 family (blue). Points show average values and error bars show root mean square deviations. Labels represent instruments applied to the generation of the Bgl2 model or PDB ID for structures of homologous glycosidases.

We performed calculations of equilibrium molecular dynamics for Bgl2p models generated by AlphaFold and SwissModel, Robetta, and Tasser servers and calculated the average entropies of torsion angles of *S_tors_* and potential energies of *E_glob_* globules from the obtained trajectories (Figure 1). At the same time, we estimated the entropy of two sets of torsion angles: Set I included only the angles *φ*, *ψ* and *ω* of amino acid residues of proteins, which describe only the conformation of the polypeptide backbone, and Set II also contained torsion angles *χ_n_*, describing the conformations of the side chains, hence the conformation of the entire protein molecule. We also performed molecular dynamics simulations of homologous β-glycosidases (Table 1, Supplemental Data S1, Figure S1) and calculated appropriate estimates that could be used as a reference when comparing Bgl2p models.

The model from i-Tasser demonstrated the most non-optimal *E_glob_*, as did the model from Robetta. The SwissModel significantly outperformed them in this regard and showed much smaller *S_tors_*. The differences in *S_tors_* and *E_glob_* between the AlphaFold and SwissModel models are comparable to the differences between homologous glycosidases (Figure 1). The AlphaFold model is characterized by the lowest entropy of torsion angles from Set I and the lowest potential energy among all the considered Bgl2p models.

We assumed that AlphaFold provides a reasonably accurate prediction of the conformation of the peptide backbone of the protein but wondered how optimal the conformation of the side groups of amino acid residues predicted by this tool was. Hence, we attempted an in-depth relaxation of our chosen Bgl2p model by means of multi-stage molecular dynamics simulations. In one stage of this relaxation, electrostatic interactions in a protein globule were first smoothly tuned off and then smoothly switched on. During this simulation, restraints were imposed on φ, ψ and ω torsion angles of the peptide backbone to prevent unfolding of the globule. We expected that in this process, suboptimal non-covalent interactions with a pronounced electrostatic component, such as hydrogen bonds present in the predicted structure, would disappear. However, the trajectory obtained following this process had worse potential energy and entropy value than the trajectory coming directly from the AlphaFold model. Therefore, this model and its initial trajectories will be analyzed below.

### 2.2. Structure and Position of Strong Amyloidogenic Sites F_83_TIFVGV_89_, N_190_AFS_193_ and G_268_VNVIVFEA_276_

**Figure 2:**
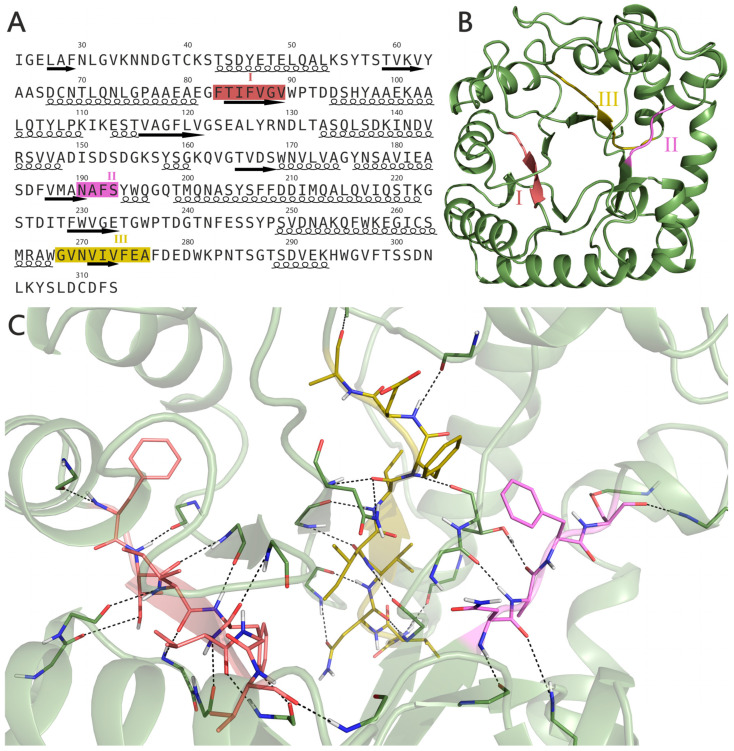
The structure of Bgl2p and position of strong amyloidogenic sites F_83_TIFVGV_89_ (Site I) are highlighted in coral, N_190_AFS_193_ (Site II)—in purple and G_268_VNVIVFEA_276_ (Site III)—in olive. Primary structure of Bgl2p with designations of the secondary structure elements (**A**): α-helices underlined as helices, β-strands underlined as arrows. Tertiary Bgl2p structure (**B**) and the part of a molecule with sites I, II and III (**C**). The black dashed lines show hydrogen bonds between amyloidogenic sites and the rest of the Bgl2p globule, shown by green tubes and ribbons. Oxygen, nitrogen and hydrogen atoms are shown as red, blue and grey sticks, correspondingly.

According to our proposed model, the Bgl2p globule is arranged as follows. Its core consists of a distorted β-barrel, which is surrounded by α-alpha helices. Each sheet of the β-barrel has its own α-helix connected to it by a loop. Previously, the presence of three strong amyloidogenic sites in Bgl2p was predicted by computational approaches [11] (Figure 2A). The analysis of elements of the secondary structure reveals that all three amyloidogenic sites, to some extent, adopt conformations corresponding to the β-sheet (Figure 2B). It is, therefore, necessary to consider the structural arrangement of these regions in more detail.

Site I makes up one of the clapboards of the central β-barrel—all its residues, with the exception of disordered Phe-83, are stacked in a β-sheet. This observation is supported by an analysis of the hydrogen bonds of this site (Table S1), most of which are formed by it with neighboring elements of the β-barrel in a manner characteristic of the β-sheet. The exceptions include the hydrogen bonds between the side chain of Thr-84 and the adjacent loop I_111_KESTVA_117_, as well as the hydrogen bond of Phe-83 with the preceding α-helix T_70_LQNLGPAAEA_80_.

Site II is the shortest. It is located on the boundary of one of the β-sheets of the core of the Bgl2p globule and a long loop connecting it to the subsequent α-helix M_200_QNASYSFFDDIMQALQVIQSTK_222_. Site II begins with the Asn-190 residue having a β-sheet conformation, then continues with disordered residues Ala-191 and Phe-192 and ends with the Ser-193 residue in the conformation corresponding to β- turn around. However, the hydrogen bonds of Ala-191 are similar to those formed in the β-sheet. The Ser-193 side group forms a hydrogen bond with a subsequent α-helix (Table S1).

Site III, like Site I, participates in the central β-barrel, albeit with less order. Its residues N_270_VIV_273_ are stacked in a β-sheet, the residues Val-269 and Glu-275 are disordered, Gly-268 forms a turn, and the residues Phe-274 and Ala-276 have a β-turn conformation. As in the case of Site I, the hydrogen bonds of Site III (Table S1) are characteristic of the β-sheet spatial arrangement.

Site I is firmly embedded within the core of the Bgl2p structure and, therefore, is unlikely to participate in the formation of amyloid. This statement will be valid for Site III, although to a lesser extent (Figure 2C). Site II differs from Sites I and III as it has a less ordered structure. As a result, it is more flexible and, therefore, more likely to adopt a conformation that leads to amyloid formation. At the same time, Site II is placed more closely on the surface of the Bgl2p globule than Site I or Site III, which makes it much more accessible for participation in amyloid interactions, for example, when the conformation of a protein, possibly changes as it leaves the cell wall during biofilm formation or during the development of candidiasis in the mammalian body.

Thus, Site II is, in all probability, the only strong amyloidogenic site that, under physiological conditions, but evidently not in CW, can participate in the process of protein fibrillization. Nevertheless, the other two sites, I and III, as well as Site II, might participate in varying degrees in the formation of amyloid fibrils after protein extraction under heating. The multiple sequence alignment of Bgl2p homologs demonstrates relatively low conservation for amyloidogenic peptide I and relatively higher conservation for peptides II and III (Supplemental Data S2, Figure S2).

**Figure 3:**
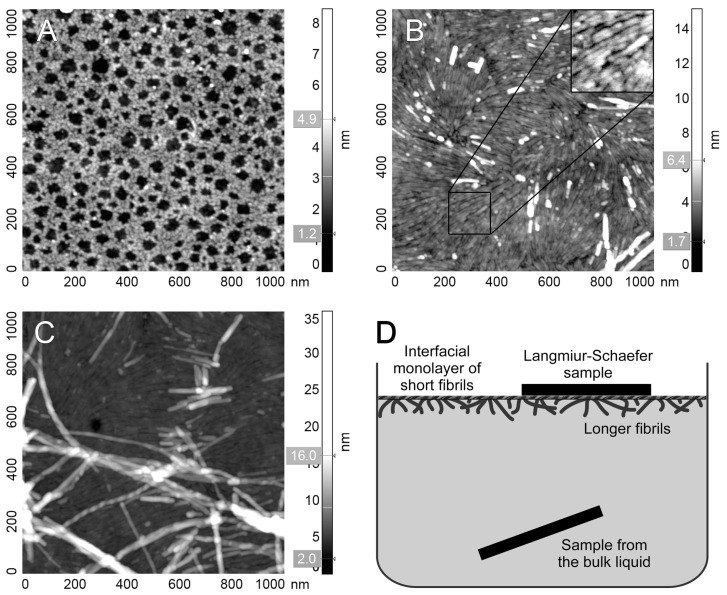
Representative AFM images of V_187_MANAFSYWQ_196_ peptide on Ultraflat polystyrene support. (**A**) Sample from the bulk liquid. (**B**,**C**) The sample was prepared by Langmuir–Schaefer and transferred from the liquid-air interface. Panel B illustrates the complete monolayer of short fibrils present on the liquid-air interface with rare fibrils on top (white). The inset contains 2× enlarged fragment of short fibrils monolayer for visual clarity. Panel C hows bundles of longer fibrils of various morphology on top of this monolayer. (**D**) The scheme of prepared samples revealed the localization of fibrils.

It was demonstrated earlier [11] that the peptide V_187_MANAFSYWQ_196_ containing Site II can fibrillate with the formation of amyloid structures binding ThT. In this study, the morphology of fibrils formed by this peptide was characterized using atomic force microscopy. In a bulk liquid, no fibrils were found (Figure 3A). Instead, the image contains small globular objects with an apparent height of 2.2–2.4 nm, which can be attributed to nonfibrillar peptide oligomers. On the other hand, a Langmuir-Schaefer transfer from the liquid-air interface resulted in a complete monolayer consisting of short fibrils (Figure 3B). On top of this monolayer, bunches of longer fibrils of various morphology (straight and spiral ones) were found (Figure 3C). The absence of these long fibrils on samples from the bulk liquid infers that the interfacial monolayer of short fibrils acts as a seed for long fibrils formation (Figure 3D). To conclude, these data are direct evidence of the amyloidogenic nature of this peptide. Moreover, it is capable of forming multiple types of fibrillar structures.

Due to the localization of amyloidogenic sites I, II and III in the Bgl2p globule, its participation in its attachment in the CW is doubtful. However, Site II can probably play a role in the amyloidization of Bgl2p when it enters the body of higher eukaryotes or during the formation of biofilms—that is, in case of changing of the native Bgl2p fold in one way or another. The amyloidogenic potential of Site II demonstrated here and its significance for medicine should be studied separately.

### 2.3. Conformation of the C-Terminal Region

As the modeling did not reveal an obvious role of strong amyloid sequences of Bgl2p in the attachment in CW by forming amyloid structures, we investigated the C-terminal region of this protein. This structure has not been sufficiently studied to date. However, the deletion of this region caused this protein to completely lose both the ability to attach in CW and to form fibrils in the environment [19].

The Bgl2p structure contains a disordered C-terminal region G_296_VFTSSDNLKYSLDCDFS_313_. In the proposed model, this part of the peptide chain is in contact with an α-helix formed by residues from Val-250 to Trp-267. In this position, it is stabilized by a fairly strong stacking interaction between the Phe-298 residue belonging to the C-terminal site and the Phe-277 residue. Also, the residues of Phe-298 and Lys-305 of CTR form stable hydrogen bonds with the rest of the protein (Table S2). The analysis of the secondary structure of CTR revealed that Ser-300 and Ser-301 residues form a turn, followed by the residues of Asp-302, Lys-305 and Tyr-306, which have conformations consistent with a β-turn, and the residues of Phe-298 and Leu-304 form a β-bridge. The remaining amino acid residues of CTR do not form elements of the secondary structure. Overall, the stability of CTR is supported by five strong hydrogen bonds (Table S2), as well as a disulfide bridge between residues Cys-262 and Cys-310, which was predicted by the AlphaFold program and is probably the main factor supporting CTR structure described above. At the same time, these amino acid residues are quite conservative for a number of Bgl2p homologs in representatives of the Ascomycota group (Supplemental Data S2, Figure S2).

Three algorithms predicted the disordered C-terminal region of Bgl2p as potentially amyloidogenic [11]. The ability of the C-terminal region to participate in amyloid formation is supported by the following considerations. Firstly, it is located on the surface of the globule. Secondly, the residues of these sequences do not form hydrogen bonds or stacking interactions with the rest of the globule, which simplifies their reorientation to another peptide chain. However, the amyloidogenic potential of these sequences is counteracted by the presence of a disulfide bridge between Cys-262 and Cys-310, which helps to anchor the C-terminal region to the Bgl2p globule. However, experimental data often reveal the absence of this disulfide bridge. More detailed data on disulfide bridges are provided in Supplemental Data S3.

Using the CORDAX server [36] to predict the ability of proteins to form amyloid fibrils and predict the structure of such fibrils, the C-terminal region is not identified as amyloidogenic. However, for sites 304–309, the so-called Cordax Score (a measure of the site’s ability to form amyloid-type fibrils) has a value of ~0.52 (when an amyloidogenic site is considered to have a value of 0.61 or higher) (Supplemental Data S4, Figure S3).

Therefore, although it was not directly demonstrated that the C-terminal region of Bgl2p plays a direct role in the formation of amyloid fibrils from its molecules, the potential involvement of the C-terminal region in such interactions cannot be excluded (though not to the same extent as in typical amyloidogenic proteins).

**Figure 4:**
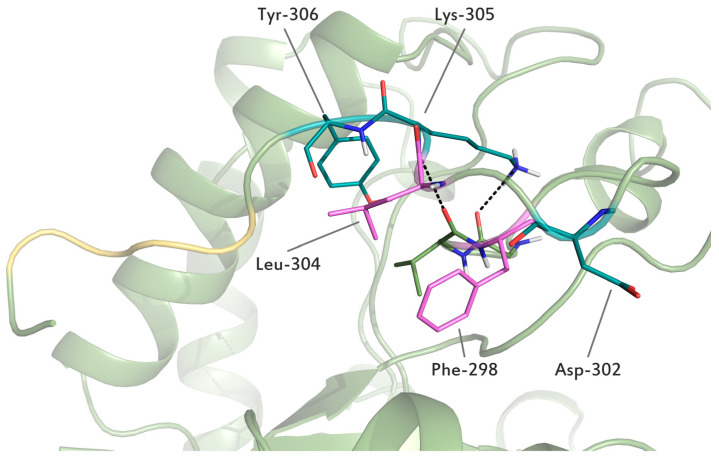
Conformation of the C-terminal region of the Bgl2p structure model. The L_308_DCD_311_ residues of the C-terminal region, forming unstable β-turn, are shown by a yellow tube. Phe-298 and Leu-304 residues, forming a stable β-bridge, are shown by violet rods, while Asp-302, Lys-305 and Tyr-306, forming stable β-turn, are shown by deepteal rods. The black dashed lines show hydrogen bonds. The laying of the rest of the globule is shown by green tubes and ribbons. Oxygen, nitrogen and hydrogen atoms are shown as red, blue and grey sticks, correspondingly.

The data from our molecular dynamics simulations regarding the conformation of the C-terminal region are not inconsistent with the previously published Bgl2p model created using the SwissModel tool [19]. Amino acid residues Asp-302, Lys-305, Tyr-306 and L_308_DCD_311_ in the AlphaFold model form β-turns, stable and unstable, consequently (Figure 4).

Since the key role of the C-terminal region in the anchoring of Bgl2p [19] and its ability to fibrillate in the environment [11,19] has been experimentally demonstrated, we supposed that the conformational changes occur in the Bgl2p molecule after deletion of the C-terminal region.

### 2.4. The Effect of C-Terminal Region Deletion on the Conformational Dynamics of Bgl2p

To investigate how the deletion of this region may affect the protein’s conformation and provide a possible explanation for the observed experimental findings, we have conducted comparative molecular dynamic modeling (see methods) for wild-type protein (Bgl2p) and a protein with nine amino acids deletion of the C-terminal region (Bgl2pΔC_305–313_).

**Figure 5:**
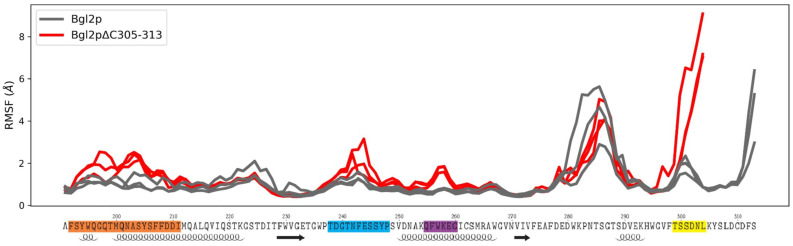
The impact of deletion of nine amino acid residues of Bgl2p from the C-terminus on the conformational stability of certain regions of the Bgl2p molecule. RMSF profiles in the C-terminal region along the sequence for Bgl2p and Bgl2pΔC_305–313_ are shown with designations of the secondary structure elements: α-helices underlined as helices, β-strands underlined as arrows. Three curves for each system correspond to three independent calculations. The protein sites with mobility changes reproducibly are highlighted in color: region 192–211—orange, region 238–248—blue, region 255–260—purple, region 299–304—yellow.

The analysis of the mobility and changes in the mobility of individual protein regions was performed by calculating the root mean square deviation profiles of C alpha atoms (RMSF). Figure 5 presents the RMSF profiles along the amino acid sequence of the C-terminal region of Bgl2p for all calculations. Using these profiles, regions of the protein that increased their degree of dynamic mobility during the removal of the C-terminal region were identified.

**Figure 6:**
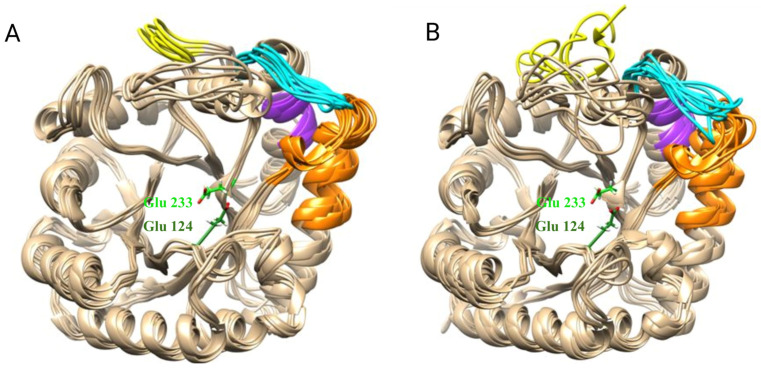
An illustration of the conformational mobility obtained during the MD calculations of (**A**) Bgl2p and (**B**) Bgl2pΔC_305–313_. The color scheme of the sites, the mobility of which changes with the deletion of the C-terminal region, corresponds to Figure 5; the amino acid residues of the active center are marked in green (Glu-233—light green, Glu-124—dark green). An overlay of several frames from the MD trajectory is presented to illustrate conformational mobility/variability.

It has been demonstrated that the deletion of nine amino acid residues from the C-terminal region of Bgl2p results in increased mobility of several sites: region 192–211, which includes a portion of a large peripheral α-helix; region 238–248, a loop that is involved in the formation of the substrate-binding region; and region 255–260, π-helix; region 299–304, the C-terminal region, is also affected (Figure 5). The comparative images of the dynamic behavior of the two modeled systems are presented in Figure 6A,B. The molecular mechanism of changing the RMSF of these sites is described in Supplemental Data S5, Figure S4.

Region 238–248, which increases its mobility during C-terminal region deletion, forms a loop that connects the penultimate α-helix with the penultimate β-strand (Figure 6).

**Figure 7:**
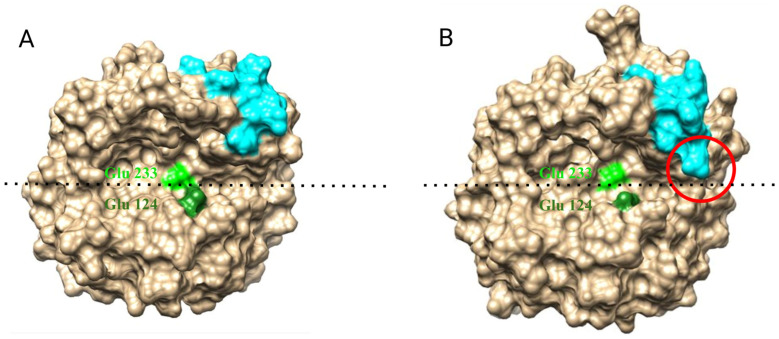
The conformations of the loop forming by aa 238–248 affect the geometry of the Bgl2p catalytic gap. (**A**) Bgl2p structure. (**B**) Bgl2pΔC_305–313_ structure. Loop 238–248 are depicted in cyan. Active site: Glu-233—light green, Glu-124—dark green. The dotted line indicates the catalytic gap. The red circle indicates the overlapping zone of the catalytic gap.

Analysis of the protein’s surface in various conformational states reveals (Figure 7) that this loop is located near the catalytic cleft and may influence its geometry and, consequently, the protein’s interaction with the substrate. Figure 7A,B illustrates the various conformations of the loop and its effect on the accessibility of the active site. More detailed data on conformations of loops and active sites is in Supplemental Data S6 (Figure S5, Table S3) and S7 (Figure S6, Table S4).

**Figure 8:**
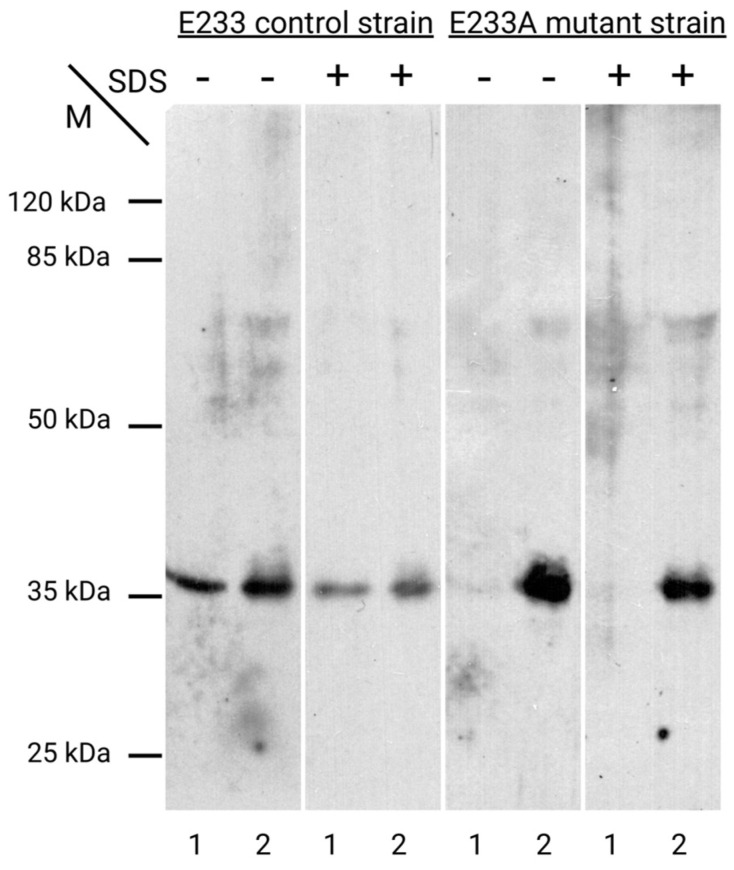
Western blot stained with antibodies to Bgl2 of samples obtained from *Saccharomyces cerevisiae* cell walls of E233 control and E233A mutant strains, untreated (-) or pretreated (+) with 1% SDS, and then incubated with 0.1 M Tris. The remaining protein was extracted from CW into a Laemmli buffer with β-mercaptoethanol after Tris incubation (lanes 1). Proteins extracted in Tris (lanes 2). All extracts were equalized by the optical density of untreated CW.

It should be noted that the destabilization of the sites forming the loop can lead to the shielding of residues that form the active center of the enzyme. Experimental data indicate that the area of the active center represents another site of the protein that can be involved in its attachment within the thickness of the glucan matrix of the cell wall (Figure 8). It can be observed that CW of Glu-233/Ala mutant strain (E233A) has practically no Tris-non-extractable Bgl2p. Based on the results, it can be concluded that the replacement of the catalytic amino acid residue Glu-233 with Ala leads to a significant reduction in the strength of protein attachment in the CW compared to the wild type (Figure 8).

Summarizing we can conclude that experimental data indicating that the active center possibly participates in the anchoring of Bgl2p in the cell wall correlated with modeling: deletion of CTR leads to the conformational change of loop 238–248 and the covering of the catalytic cleft. It is possible that shielding of the catalytic cleft does not allow this region of the molecule to participate in the anchoring on glucan. However, disruption of the active center structure does not lead to a complete loss of Bgl2p anchoring. At the same time, C-terminal deletion results in a complete loss of the ability of Bgl2p to be strongly attached in the CW. It can be concluded that with the deletion of CTR, the Bgl2p molecule loses an additional site possibly involved in anchoring.

Here, it is important to take into account two facts: Bgl2p is characterized not only as a glucan-binding enzyme without a carbohydrate-binding module [5] but also it cannot attach to CW in the absence of cell wall GPI-proteins [26].

With the deletion of the sequence 305–313, Bgl2p loses its ability to attach to CW because of the loss of two opportunities to anchor: through β-turns and the catalytic cleft, respectively. The elimination of these sites simultaneously, which occurs when the C-terminal region is deleted, maybe the reason for the complete loss of the possibility of Bgl2p attachment to the cell wall [19]. It should be noted that in the presence of a CTR in the Bgl2p molecule, the protein demonstrates the ability to fibrillate after its release from the CW into the culture medium [11]. It is obvious that with the loss of CTR, the Bgl2p molecule loses not only the sites for the cell wall attachment but also machinery that promotes fibrillization after leaving the CW. We hypothesize that such a mechanism may be a combination of the amyloidogenic activity of the C-terminal weak amyloidogenic region with structural features of β-turns recently described to be involved in the fibrillization process [37,38]. To realize this mechanism, the mobility of the C-terminus is required not only in the region D_311_FS_313_ (Figure 5, gray lines) but also in the region G_296_VFTSSDNLKYSLDCDFS_313_. In the presence of Cys-262–Cys-310 disulfide bond, predicted by Alphafold, this region does not possess the required mobility.

**Figure 9:**
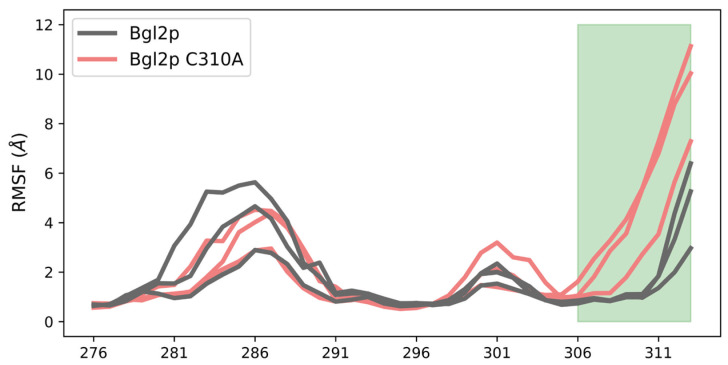
The conformational mobility of the C-terminus (marked in green) of Bgl2p structures, non-mutated (gray) and with C310A substitution (coral). RMSF profiles of three trajectories for both structures.

According to LC-MS/MS analysis under non-reducing conditions, Cys-310 was determined as dehydroalanine or unpaired cysteine in more than a third of Bgl2p samples in position 310 also even more often, Cys-262 was determined as not involved in the formation of a disulfide bond. These experimental data suggest that the disulfide bond between Cys-262 and Cys-310, predicted by Alphafold, is not always realized in vivo, and the substitution of cysteine to alanine at position 310 may be a good way (legitimate) for studying the C-terminal region mobility in the absence of a disulfide bond. The results of MD modeling demonstrated that the site 306–313 acquired greater mobility and more possible conformations in the case of Bgl2p C310A compared to Bgl2p without mutation (Figure 9, Supplemental Data S8, Figure S7).

Summarizing the data of molecular modeling and the results of experiments, it can be suggested that the C-terminal region is most important not only for Bgl2p attachment in CW but also for regulation of the process of attachment or release of this protein from the molecular complex of the cell wall. In the absence of a disulfide bond between Cys-262 and Cys-310, the C-terminus is tightly pressed against the protein globule, and the amyloid sequence, as well as the β-turn sequences, are inaccessible both for the formation of amyloid strands and for interaction with other proteins, for example, with GPI-proteins of CW. In a conformation characterized by the presence of a disulfide bond between Cys-262 and Cys-310, Bgl2p, in all probability, can leave CW for the environment under physiological conditions.

In the absence of this bond, the C-terminal region becomes more mobile. The C-terminal amyloid sequence, as well as β-turns in the absence of a disulfide bond, become available for interaction with other CW proteins. It can be assumed that the absence of a disulfide bond contributes to the process of the interaction that occurs between two or more Bgl2p molecules, leading to protein fibrillization.

Along with the active center that secures attachment of Bgl2p to its substrate, glucan, it allows the protein to anchor in CW and demonstrate the properties of a glucan-binding protein [5]. The interaction of Bgl2p with other CW proteins with participation of the C-terminal β-turns localized in this region contributes to the protein’s strong attachment in CW. The active center of the enzyme, in all likelihood, also contributes to Bgl2p anchoring in CW. All the properties of Bgl2p described here indicate that the strong amyloid peptide of its molecule and C-terminal amyloid region, together with β-turns, contribute to the interaction of the protein in the CW and in culture medium to varying degrees and, probably, in different ways. Under conditions conducive to the protein leaving CW and coming into the environment, the amyloidogenic potential of its molecule can be increased many times. Also, it should be noted that LARKS (low-complexity amyloid-like kinked segments) have been actively studied in recent years [39]. The literature presents a concept describing the role of these sequences in amyloid-related diseases. The search for LARKS in yeast cell wall proteins may be a promising direction for the detailed study. These facts require further investigation since they may have significant medical value.

## 3. Materials and Methods

### 3.1. Molecular Dynamics Simulations

We compared Bgl2p *S. cerevisiae* models built by AlphaFold [28,40,41], SwissModel [42], Robetta [43] and i-Tasser [44] servers. The template for the construction of the last three models was the homologous Bgl2p glycosidase *Rhizomucor miehei* (PDB ID: 4WTP) [32] and seven glycosidase structures from the GH17 family (Table 1).

The simulated protein globules were analyzed in dodecahedral cells with a minimum distance of 2 nm from the protein globule to the cell border, using the TIP3P water model [45] and a salt concentration of 150 mM NaCl.

Calculations of molecular dynamics and analysis of the obtained trajectories were carried out using GROMACS [46] version 2020.1. Molecular-mechanical models of amino acid residues were constructed using the AMBER-ff14SB force field [47]. The simulation was performed at a temperature of 300 K, maintained by a velocity scaling thermostat with an additional stochastic term [48] with a coupling constant of 1 ps and periodic boundary conditions with an isotropic constant pressure maintained by a Parrinello–Rahman barostat [49,50,51] with a coupling constant of 1 ps. Electrostatic interactions were processed using the PME algorithm [52] with a grid step of 0.12 nm and a fourth-order interpolation. The coordinates were recorded in the trajectory file every 10 ps, and the integration step was 2 fs. The length of each obtained trajectory was 300 ns. The covalent bonds of hydrogen atoms were constrained using the LINCS algorithm [53].

For the analysis of the trajectories, the GROMOS method [54], the DSSP program [55], and the PyMOL program were used. Calculation of the potential energy of a protein globule and entropy of its torsion angles was performed using compressibility of the discrete representation [29].

Bgl2p models with Cys-310 replaced by alanine residue (C310A) were generated based on the model generated by the AlphaFold program using the PyMOL program (Mutagenesis Wizard), after which the side chain rotameters were optimized using the FoldX program (RepeirPDB command) [56]. The simulation was carried out under the conditions described above. Three trajectories with a length of 300 ns each were obtained.

### 3.2. Atomic Force Microscopy

Two-sided sheets of Ultraflat polystyrene (UFPS) were prepared as previously described [57]. A square (25 × 25 mm, 0.3 mm thick) of polyvinyl chloride sheet was thoroughly washed with detergent, followed by MilliQ water. The sheet was spun at 2000 RPM, rinsed with ultrapure toluene, and coated with 100 g/L of polystyrene in toluene. The same procedure was repeated for the other side of the sheet. The UFPS was stored in a closed container prior to use to prevent contamination. Small pieces of the desired size were cut from UFPS for sample preparations.

A V_187_MANAFSYWQ_196_ peptide was studied in TRIS buffer (50 mM, pH 7.5) at 25 μg/mL. For preparation of the sample from the bulk liquid, UFPS was fully immersed in a pure buffer. Next, an equal volume of the peptide in double concentration (50 μg/mL) was added and mixed by gentle pipetting. The sample was incubated for 30 min; the surface of the liquid was cleaned with filter paper, and immediate sample withdrawal was performed. The sample was triple-washed with MilliQ water and dried on air. The second sample was prepared by Langmuir-Schaefer transfer from the liquid-air interface. The surface of the peptide solution was cleaned by filter paper and left for 30 min for fibril formation. The liquid surface was touched by UFPS horizontally, followed by a triple wash of the sample in MilliQ water.

Samples were examined with N’Tegra Prima scanning probe microscope (NT-MDT, Moscow, Russia) in semi-contact mode on air using silicon NSG03 cantilevers (NT-MDT, Moscow, Russia) with a tip curvature radius of 10 nm, resonant frequency of 47–150 kHz, and force constant of 0.35–6.1 N/m. Raw topography was flattened using polynomial background subtraction.

### 3.3. Yeast Strains and Cultivation Conditions

*S. cerevisiae* strain *bgl2*Δ (Mat α *his3∆1 leu2∆0 lys2∆0 ura3∆0 bgl2::LEU2*) [19], obtained from BY4742 (EUROSCARF) and its derivative obtained as described below with plasmids pEMBL-yex4-BGL2-E233A (E233A mutant strain) and pEMBL-yex4-BGL2 (E233 control strain) respectively were used. For experiments, yeast was grown in a liquid medium at 30 °C with aeration at 200 rpm until the mid-logarithmic stage. BY4742 was grown on a YPD medium. E233A and PS strains were grown on a uracil-free synthetic medium [58] supplemented with 1% D-glucose and 1% D-galactose for the moderate induction of *BGL2* under the control of the *GAL10-CYC1* hybrid promoter.

### 3.4. Construction of E233A Mutant Strain

pBGL2-WT plasmid was obtained as previously described [19]. PCR mutagenesis of pBGL2-WT plasmid was performed with a Q5 site-directed mutagenesis kit (New England Biolabs, Moscow, Russia). For the construction of pBGL2-E233A plasmid with E233A mutation 5′-GATATTACCTTCTGGGTTGGTGCGACTGGTTGGCCAACTGATGG-3′ and 5′-GGTAGAACCTTTAGTAGATTGGATAACC-3′ primers were used. Plasmids were transformed using the lithium acetate/ssDNA/PEG method [59] into *bgl2∆* strain.

### 3.5. Yeast Cell Wall Isolation and Protein Extraction

Yeast cell walls from mid-logarithmic phase yeast strains were isolated, as described earlier [25]. Then, CW was treated or non-treated with 1% SDS for 1 h at 37 °C and washed as described earlier [25]. Then, proteins from both SDS-treated and non-treated CW were extracted with 0.1 M Tris, as described earlier [25]. For the extraction of non-covalently attached CW proteins unextracted proteins (Tris CW samples), wash the cell pellet from first centrifugation with fresh 0.1 M Tris and then remove the supernatant by centrifugation at 2580× *g* for 5 min, then extract into Laemmli sample buffer with additional 5% β-mercaptoethanol and 0.625 mM EDTA.

### 3.6. Electrophoresis, Western Blot Analysis

Polyacrylamide gel electrophoresis (PAGE) and Western blot analysis were performed as described earlier [25]. For PAGE, various CW extracts were equalized by the optical density of untreated CW at 540 nm.

### 3.7. Sample Preparation for LC-MS/MS Analysis

Protein extracts in 0.1 M Tris washed with 1% SDS CW of BY4742 strain were prepared as described earlier [25]. Protein extracts in milliQ water from ones were prepared by heating of CW suspension (at the ratio 1 optical density of CW at 540 nm to 10 µL of milliQ) on a boiling water bath for 10 min. The extracts were separated from the CW by centrifugation at 12,000× *g* (Eppendorf, Moscow, Russia) for 2 min. Further sample treatment, liquid chromatography, and mass spectrometry were carried out as described previously [60] without the procedure of reduction of disulfide bonds and blocking of cysteines.

## 4. Conclusions

Thus, summing up all the experimental data and the results obtained in the process of comparative modeling, we can propose the following hypothetical scheme for the attachment of the studied protein to CW.

It is possible to suppose that in the absence of the disulfide bridge between Cys-262 and Cys-310, Bgl2p is attached to the CW in three ways:

1 With the interaction of the active center of its molecule and substrate—glucan;
2 With the interaction of β-turns located in the C-terminal region of the Bgl2p molecule and other cell wall proteins;
3 By amyloid interactions among amino acid sequences localized in the C-terminal region of Bgl2p molecules one with another, possibly enhanced by β-turns and resulting in amyloid structure formation inside the cell wall.

In the presence of the Cys262-Cys310 disulfide bond, the protein is likely to leave the cell wall and move into the medium, where it could serve as an inductor for a fibrillization of other proteins, possibly with the participation of Site II.

It was demonstrated earlier that Bgl2p can fibrillate under physiological conditions not only in the environment after entering from the cell wall but also after extraction under heating. In this work, we have expanded our understanding of the potential ability of a protein to fibrillate under different conditions and suggested that the amyloid sequences of Bgl2p involved in the process of its fibrillization have to be different in CW and in the medium as well as in physiological conditions and after heating since the conformation of the protein varies.

Direct evidence that Bgl2p can form amyloid-type fibrillar structures within the cell wall has not been obtained yet. However, there is sufficient indirect evidence to support this possibility [10,11,22,23].

It should be noted that experimental evidence confirms that amyloid structures formed by Bgl2p molecules inside CW are extremely weak (they are usually destroyed when boiled in water). Nevertheless, exposure of intramolecular amyloid amino acid sequences may lead to the manifestation of more strong Bgl2p amyloid properties. We consider the amyloid properties of this protein to be the most important for our further study, and we are currently focusing our efforts on the importance of Bgl2p for medicine.

## Figures and Tables

**Table 1:** Bgl2p homologs for which the structure has been determined experimentally.

PDB ID	Organism	Resolution, Å	Reference
3URA	*Brevundimonas diminuta*	1.88	[30]
6JMS	*Cryptomeria japonica*	1.50	[31]
4WTP	*Rhizomucor miehei CAU432*	1.30	[32]
1GHS	*Hordeum vulgare*	2.30	[33]
2CYG	*Musa acuminata*	1.45	[34]
4IIS	*Hevea brasiliensis*	2.67	[35]
4HPG		2.54	

## Data Availability

Data is contained within the article and Supplementary Materials.

## Supplementary Materials

The following supporting information can be downloaded at: https://www.mdpi.com/article/10.3390/ijms252413703/s1.
