# Supplementary material for: Yeast Glucan Remodeling Protein Bgl2p: Amyloid Properties and the Mode of Attachment in Cell Wall
Source: Int J Mol Sci. 2024 Dec 22;25(24):13703. doi: 10.3390/ijms252413703 (PMC11677059; doi:10.3390/ijms252413703)
Supplement: Supplementary file 1 [file ijms-25-13703-s001.zip › IJMS_Supplemental data.pdf]

Supplemental data

Supplemental tables.

Table S1. Hydrogen bonds of the Bgl2p amyloidogenic sites I, II and III, found in more than 50% of the trajectory frames. Residues, which do not belong to the corresponding amyloidogenic site, are highlighted in italics.

Donor	Acceptor	Occurrence, %
I amyloidogenic site		
Phe–83/N–H	*Ala*–78/O	80
Thr–84/N–H	*Ser*–58/O	86
Thr–84/O$_\beta$–H	*Ser*–114/O	69
Ile–85/N–H	*Thr*–115/O	86
Phe–86/N–H	*Val*–60/O	100
Val–87/N–H	*Gly*–118/O	72
Val–89/N–H	*Leu*–120/O	94
Val–60/N–H	Thr–84/O	96
Val–62/N–H	Phe–86/O	85
Ala–117/N–H	Ile–85/O	94
Leu–120/N–H	Val–87/O	99
Gly–122/N–H	Val–89/O	91
II amyloidogenic site		
Asn–190/N–H	*Asp*–167/O	99
Ala–191/N–H	*Glu*–233/O	99
Ser–193/O$_\beta$–H	*Ala*–203/O	86
Trp–169/N–H	Asn–190/O	77
Gln–196/N–H	Ser–193/O	80
Thr–234/O$_\beta$–H	Ala–191/O	98
III amyloidogenic site		
Val–269/N–H	*Met*–264/O	83

Val–271/N–H	*Glu*–26/O	99
Ile–272/N–H	*Val*–231/O	92
Val–273/N–H	*Ala*–28/O	98
Phe–274/N–H	*Thr*–234/O	97
Glu–275/N–H	*Trp*–295/O	78
Glu–26/N–H	Asn–270/O$_\delta$	98
Ala–28/N–H	Val–271/O	68
Asn–30/N–H	Val–273/O	95
Asn–30/N$_\gamma$–H	Val–273/O	90
Val–231/N–H	Asn–270/O	99
Phe–298/N–H	Ala–276/O	96

Table S2. Hydrogen bonds of the Bgl2p C-terminal region, found in more than 50% of the trajectory frames. Bonds between residues of the C-terminal region are denoted as internal, bonds between the C-terminal region and the rest of the protein are denoted as external. Residues, which do not belong to the C-terminal region, are highlighted in italics.

Donor	Acceptor	Occurrence, %
	Internal	
Thr–299/N–H	Asn–303/O	90
Thr–299/O$_\beta$–H	Asn–303/O	55
Asp–302/N–H	Thr–299/O	70
Lys–305/N–H	Val–297/O	94
Lys–305/N$_\varepsilon$–H	Gly–296/O	84
	External	
Phe–298/N–H	*Ala*–276/O	96
Lys–305/N$_\varepsilon$–H	*Thr*–238/O$_\beta$	80

Supplemental data S1.

Analysis of the structure of Bgl2p and experimentally determined structures of its homologues.

Structural alignment of Bgl2p homologs with a known structure was constructed using PyMOL program. For comparison of these proteins and the Bgl2p structure constructed by the AlphaFold program, the TM-score was calculated using the server (https://zhanggroup.org/TM-score/) (Figure S1).

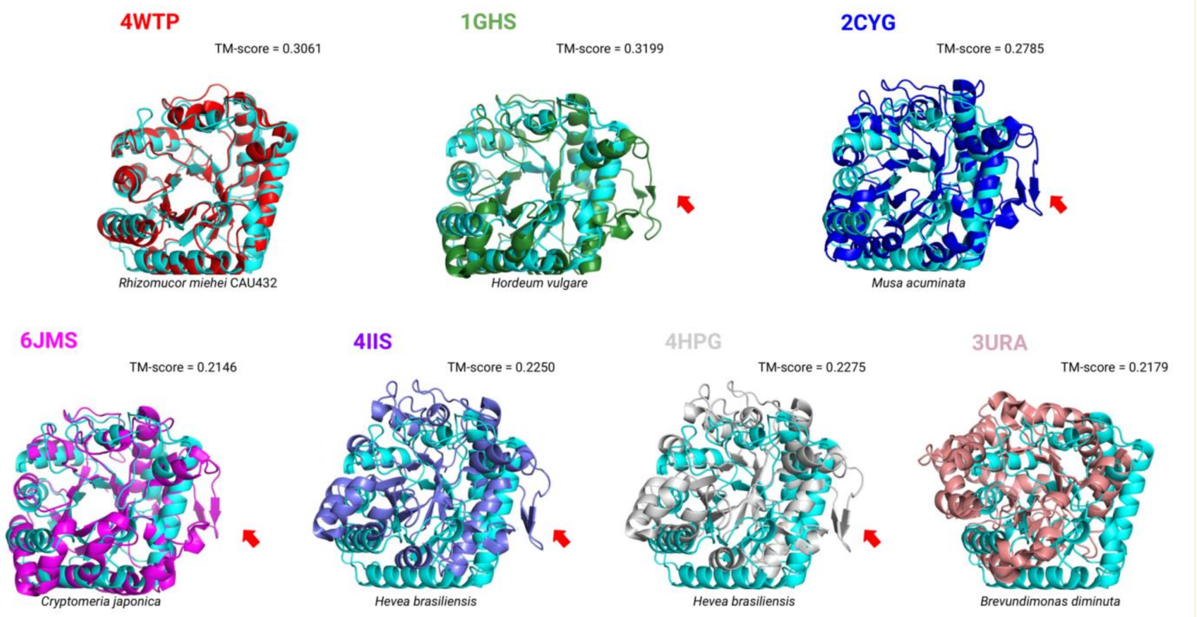

Figure S1. Structural alignment of Bgl2p (shown in cyan) and homologues with experimentally determined structures. The PDB ID is shown in the figure in the same color as the protein structure. For each protein, the TM-score relative to the Bgl2p structure is indicated. The red arrow indicates a loop with a beta turn in its composition in the same part of the protein for structures 1GHS, 2CYG, 6JMS, 4IIS and 4HPG.

The greatest structural similarity to Bgl2p was observed for the 4WTP and 1GHS proteins. In general, the characteristic type of TIM barrel folding for proteins of the GH17 family was preserved in each of the structures. It is noteworthy that there is a loop with a beta turn in its composition in the same part of the protein for structures 1GHS, 2CYG, 6JMS, 4IIS and 4HPG that are present in plants. At the same time, Bgl2p, 4WTP (proteins from organisms of the Fungi group) and protein 3URA from bacterial organism do not have this loop (Figure S1).

Supplemental data S2

Construction of multiple alignment for Bgl2p homologues.

Using Protein Blast, 12 Bgl2p homologues were selected from representatives of the Ascomycota group. The selected sequences were aligned using the Clustal Omega server (https://www.ebi.ac.uk/jdispatcher/msa/clustalo), then using the pytexshade package (https://github.com/intbio/pytexshade) multiple alignment and logo plot were visualized for the C-terminal part of the alignment. There is a high degree of conservation of cysteines corresponding to Cys –262 and Cys –310 in Bgl2p, which also indicates the probability of formation of a disulfide bond between these residues. This analysis clearly visualizes AlphaFold's predictions (although the AlphaFold program makes a similar conclusion with greater reliability by using coevolution data). The presented multiple alignment shows that the Bgl2p protein is quite conserved among the Ascomycota group (Figure S2A). At the same time, despite the presence of some variable regions, amino acid residues homologous to Glu –124 and Glu –233 (amino acid residues of the active center of Bgl2p) are preserved for all selected sequences, which may indicate the preservation of the enzymatic function (Figure S2A). Our multiple sequence alignment of Bgl2p homologs demonstrates relatively low conservation for amyloidogenic peptide I and relatively higher conservation for peptides II and III (Figure S2A). In this regard, it can be assumed that the ability to form amyloids in Bgl2p homologues for peptides II and III is potentially higher relative to peptide I. Also, using the server http://imed.med.ucm.es/Tools/sias.html, the average percentage of similarity (mean = 74.5555%, stddev = 11.4311%) and the average percentage of identity (mean = 64.67%, stddev = 11.3374%) were calculated for this alignment; the BLOSUM62 matrix was selected for the calculation. The calculated percentages of similarity and identity within such a large taxonomic group as Ascomycota may indicate a relatively high degree of conservation for Bgl2p.

The ConfSurf server was also used to analyze the conservation of Bgl2p (https://consurf.tau.ac.il/consurf_index.php). The predictions of this server are consistent with the above findings: Cys –262 and Cys –310, as well as Glu –124 and Glu –233, are shown to be conservative. The predictions of this server are consistent with the higher conservation of amyloidogenic regions II and III relative to the more variable amyloidogenic region I (Figure S2B). It is noteworthy that amino acids in the region of the catalytic cleft of Bgl2p show high evolutionary conservation, which may indicate the conservation of the enzymatic function of Bgl2p (Figure S2B).

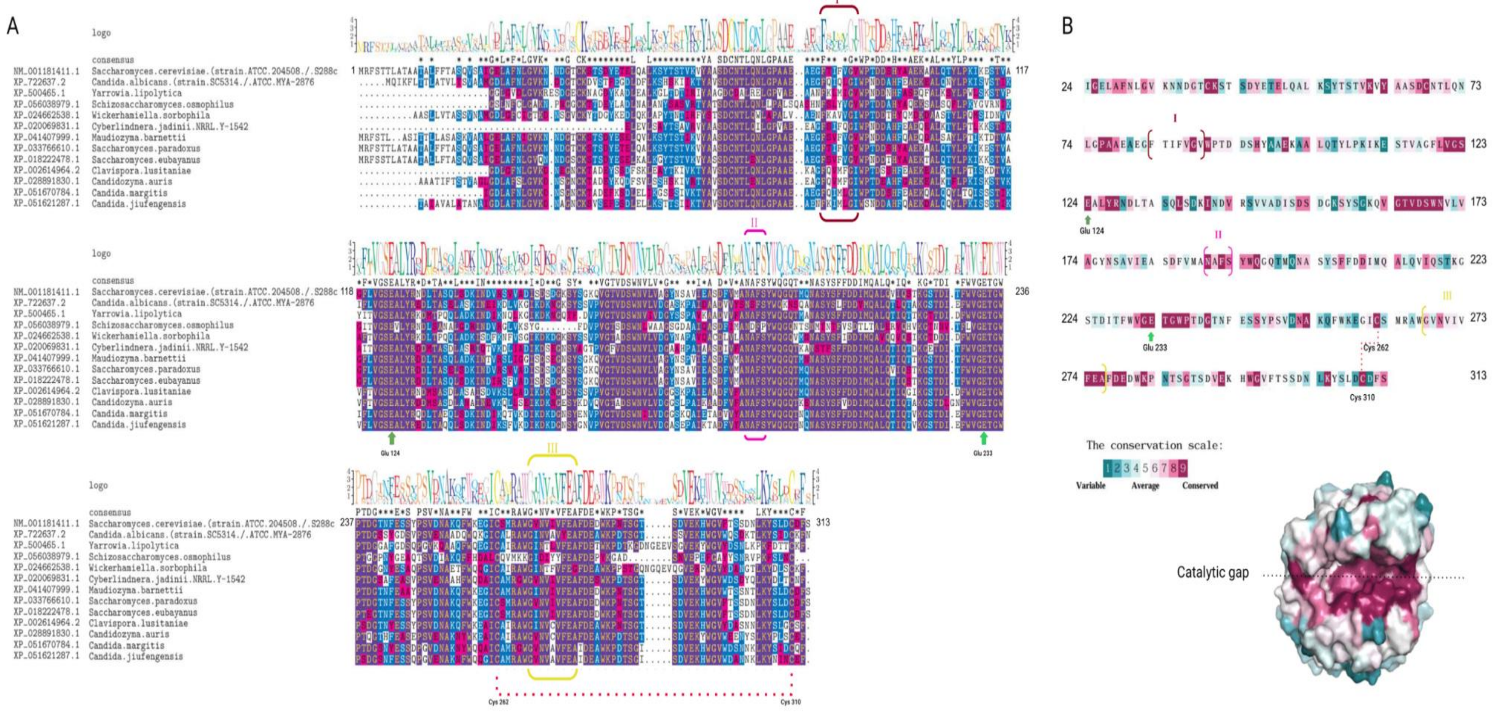

Figure S2. Analysis of Bgl2p conservation. A) Multiple alignment of Bgl2p homologs. Amyloidogenic peptides I, II and III are indicated in the figure. The amino acid residues of the active site of Bgl2p are marked with arrows (Glu –124 dark green, Glu –233 light green). Disulfide bond forming Cys –262 and Cys –310 are indicated by the orange dotted line. B) Result of ConfSurf server prediction for studying protein conservation for Bgl2p. The bottom of the panel shows the structure of Bgl2p, colored according to the degree of conservation of amino acid residues, the catalytic gap is indicated by a black dotted line. Amyloidogenic peptides I, II and III are indicated in the figure. The amino acid residues of the active site of Bgl2p are marked with arrows (Glu –124 dark green, Glu –233 light green). Disulfide bond forming Cys –262 and Cys –310 are indicated by the orange dotted line.

Supplemental data S3.

Disulfide bridges.

The Bgl2p polypeptide chain contains four cysteine residues: Cys–40, Cys–68, Cys–262 and Cys–310. In the proposed model, these residues form disulfide bridges that help to maintain the structure of the protein globule. Residues Cys–40 and Cys–68 are linked together by a disulfide bridge loops containing N_{35}NDGTCKS_{42} and V_{62}YAASDCN_{69}. It should be noted that this disulfide bridge is present both in the structure of homologous glycosidase *R. miehei* (PDB ID: 4WTP) and in the SwissModel model based on it. This suggests that the presence of this bridge is due to the convergence of loops containing cysteine residues, which is supported by interactions between the corresponding β–sheets of the central β–barrel.

The disulfide bridge between Cys–262 and Cys–310 residues braces the C–terminal section of Bgl2p to the globule. At the same time, in the SwissModel model, the conformation of the C–terminal region is different, and the cysteine residues are so significantly separated that there is no disulfide bridge between them. Is this disulfide bridge really present in the Bgl2p structure? This statement is opposed by the fact that the residue Cys–310 is located at the very end of the disordered, and therefore rather mobile, C–terminal region. However, its existence is supported by the presence of a disulfide bridge between the corresponding cysteine residues in the structure of homologous glycosidase *R. miehei*. It should also be noted that Bgl2p is an extracellular protein, and extracellular proteins are characterized by having cysteine residues that are typically oxidized. The convergence of cysteine residues Cys–262 and Cys–310 during the movement of the C–terminal region is quite possible/likely. Furthermore, it is essential to consider the characteristics of modeling the three-dimensional structure of Bgl2p using available tools In the course of work, the AlphaFold program generates a multiple alignment of amino acid sequences of homologous proteins, based on which the coevolution matrix can be calculated [28]. Therefore, AlphaFold's prediction of a disulfide bridge between Cys–262 and Cys–310 indicates that closely located cysteine residues often occur in homologous positions. On the contrary, the Swiss Model program does not utilize coevolutionary data and generates a model based on a single selected template, which makes the prediction of disulfide bridges less reliable.

Supplemental data S4.

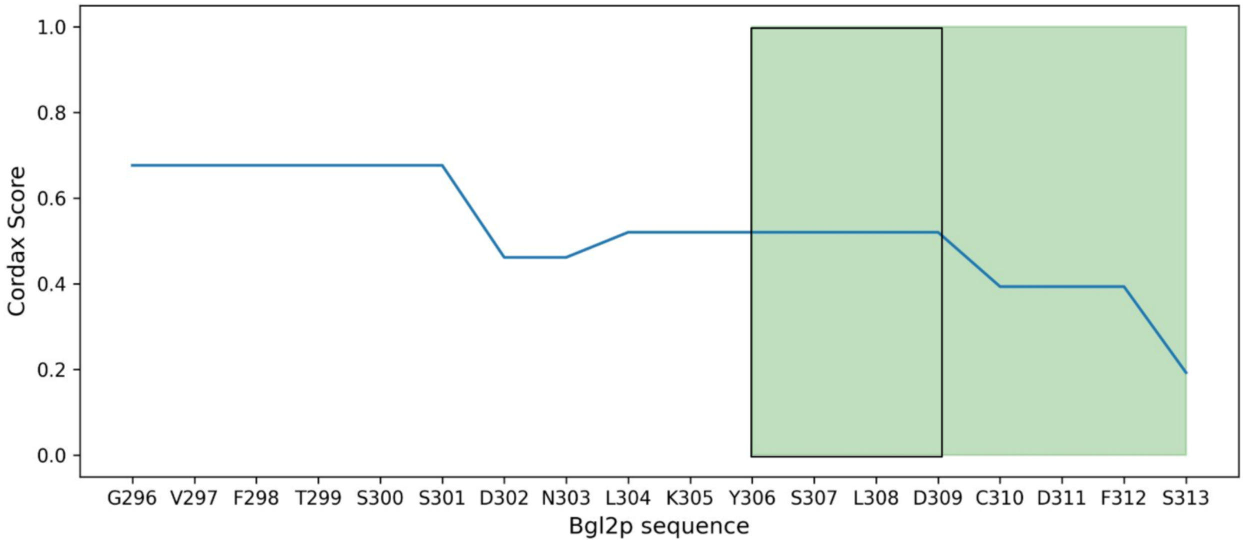

Figure S3. Cordax Score values for C-terminal region of Bgl2p 296-313. Site 306-313 is marked in green – its dynamism increases when replacing C310 with A, for section 306-309 the value is ~ 0.52 (highlighted with a black border).

Supplemental data S5.

The effect of C-terminal region deletion on the conformational dynamics of Bgl2p.

To understand the molecular mechanisms of destabilization, we analyzed the interactions between amino acid residues at different protein sites that change their mobility during C-terminal region deletion (Figure S4).

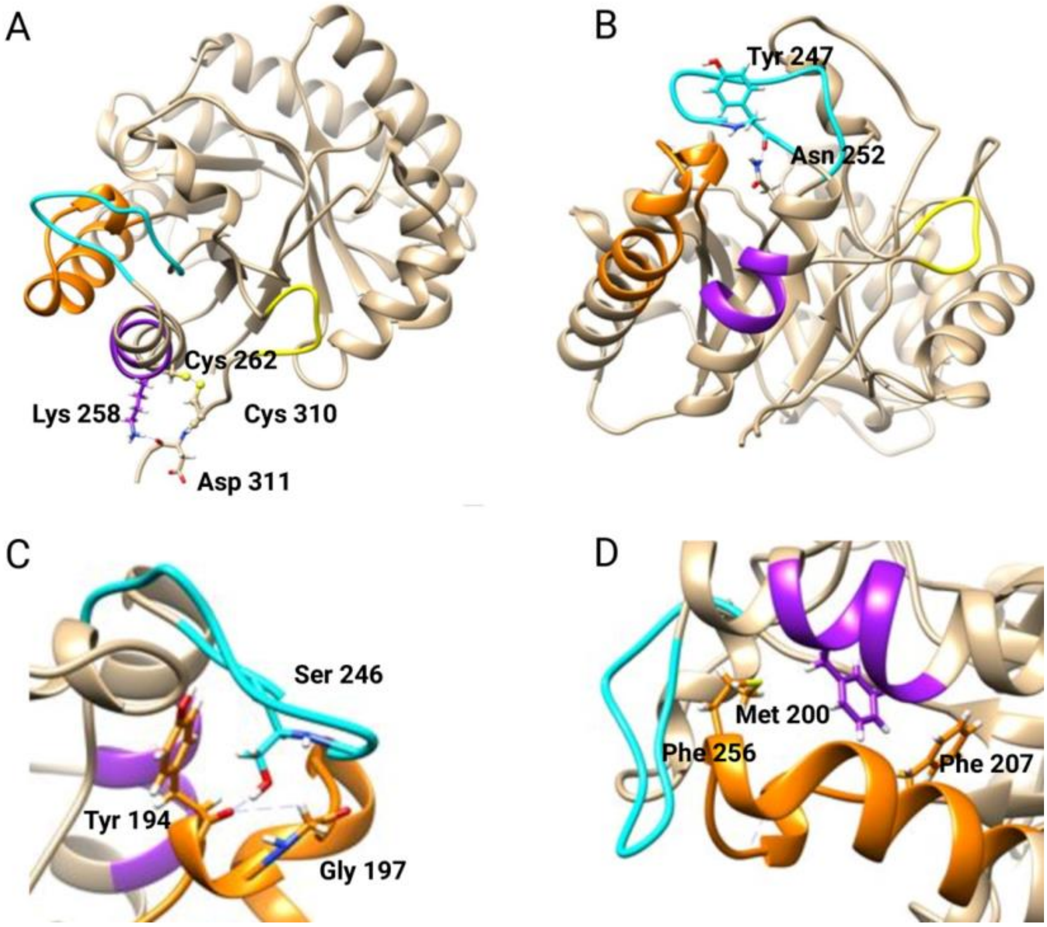

Figure S4. Interactions between sites that increase their mobility when cutting off the C-terminal, in the Bgl2p composition: A) Interactions between the cut-off C-terminal and site 255-260; B) Interaction between site 238-248 and site 255-260; C) Interaction between site 192-211 and site 238-248; D) Interaction between site 192-211 and site 255-260.

Based on the analysis, it can be hypothesized that the reason for the increased mobility of site 192-211 and site 255-260 is the loss of contacts between the C-terminal region and site 255-260, which ensure its stability. This contact which is important for stabilizing site 255-260 is the

disulfide bond between Cys-310 and Cys-262, as well as the hydrogen bond between the carbonyl oxygen Asp 311 and the amino group Lys 585 (Figure S4A). The destabilization of site 255-260 may contribute to an increased mobility in site 192-211 and site 255-260, since the amino acid residues are close to site 255-260 and its components interact with sites 192-211 (Figure S4B) and 255-260 (Figure S4D). At the same time, during the destabilization of site 192-211, there might be a loss of its interaction with site 255-260 (Figure S3C), which may also contribute to its destabilization. At the same time, the destabilization of site 192-211 may be related to a loss of interaction with site 255-260.

Supplemental data S6.

Conformation of loops.

Loop I is composed of the sequence $E_{279}DWKPNTSGTS_{289}$ (Figure S5) and originates from the amyloidogenic Site III, located in close proximity to it.

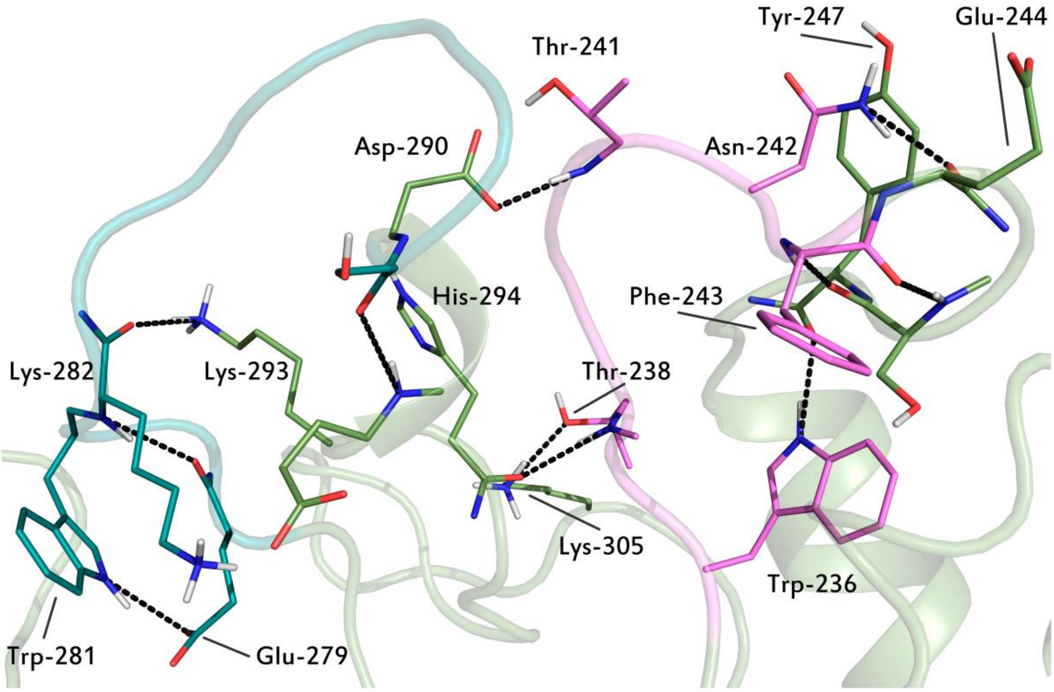

Figure S5. Loop conformation in the Bgl2p glycosidase structure model. Loop I is shown with blue sticks and cartoons; Loop II is shown with purple sticks and cartoons. The laying/structure of the rest of the globule is shown by green cartoons. The black dotted line shows hydrogen bonds.

A significant part of the loop is unstructured consisting of the residues Glu–279, Pro–283, Asn–284 and Thr–285. The residues Asp–280 and Trp–281 are of a rotational conformation, whereas Lys–282, Ser–286, Gly–287, Thr–288 and Ser–289 tend to adopt a β–rotation/turn. The

residues of Loop I form a few hydrogen bonds of moderate stability; and its structure is supported by hydrogen bonds between the residues of Glu–279, Trp–281 and Lys–282 (Table S3). The low ordering of this loop allows us to suggest that under any stressful conditions affecting the vital activity of yeast, conformational changes may occur there, leading to the exposure of the amyloidogenic Site III on the surface of the protein globule. In this case, it may be able to participate in the formation of amyloid fibrils. However, this hypothesis could be challenged by the fact that, as mentioned above, amyloidogenic Site III is involved in the formation of the central β–barrel structure in yeast.

Table S3. Hydrogen bonds of the Bgl2p loops I and II, found in more than 50% of the trajectory frames. Bonds between residues of the loop are denoted as internal, bonds between the loop and the rest of the protein are denoted as external. Residues, which do not belong to the loops, are highlighted in italics.

Donor	Acceptor	Occurrence, %
	I loop	
	Internal	
Trp–281/N_1–H	Glu–279/O_δ	53
Lys–282/N–H	Glu–279/O	79
	External	
Glu–292/N–H	Ser–289/O	75
Lys–293/N_ε–H	Lys–282/O	52
	II loop	
	External	
Trp–236/N_1–H	*Tyr*–247/O	87
Thr–238/N–H	*His*–294/O	96
Thr–241/N–H	*Asp*–290/$O_{\gamma'}$	51
Asn–242/N_γ–H	*Glu*–244/O	64
Phe–243/N–H	*Ser*–246/O	79
Ser–246/N–H	Phe–243/O	89
Lys–305/N_ε–H	Thr–238/O_β	80

Loop II, which is formed by the amino acid sequence W236PTDGTNF243, is more structured than Loop I. The residues Thr–238, Asp–239, Gly–240 and Thr–241 are stacked in a β–

turn, while theresidue Phe–243 forms a β–bridge. The residues Trp–236, Pro–237 and Asn–242 are unstructured. The laying of Loop II is supported by hydrogen bonds with other parts of the protein globule (Table S3). The residues Glu–279 and Arg–282 from Loop II are involved in the formation of the surface of the catalytic pocket. It is possible that the unstructured nature of this loop and its high conformational flexibility explain the ability of Bgl2p to exhibit both glycanosylhydrolase and glycanosyltransglycosylase activity [7].

Supplemental data S7.

Conformation of catalytic residues of glutamate.

According to the study [32], glutamic acid residues corresponding to residues Glu–124 and Glu–233 in Bgl2p (Figure S6) are responsible for the hydrolase activity of this enzyme.

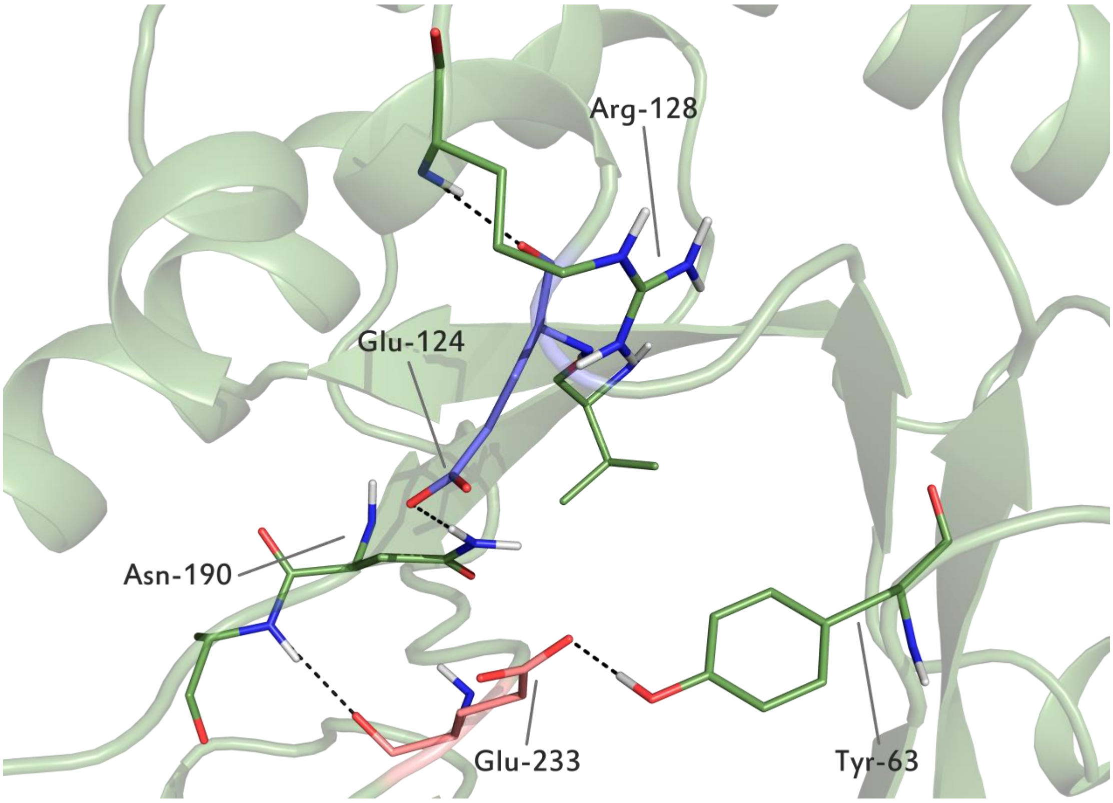

Figure S6. Conformation of the catalytic residues of glutamic acid in the Bgl2 glycosidase structure model. The residue of Glu–233 is shown with pink rods and bundles, the residue of Glu–124 is shown with blue rods and bundles. The laying of the rest of the globule is shown by green bundles. The black dotted line shows hydrogen bonds.

The Glu–124 residue is located at the end of one of the β–sheets of the central β–barrel and has a β–rotation/turn conformation. Its side chain is oriented towards Glu–233 and is stabilized in this position by a hydrogen bond with Asn–190. The Glu–233 residue is located in one of the β–sheets that make up the central β–barrel of Bgl2p and has the corresponding conformation. Its side chain is oriented towards Glu–124 and is stabilized in this position by a hydrogen bond with Tyr–63 (Table S4).

Table S4. Hydrogen bonds of the Bgl2p catalytic residues of glutamic acid, found in more than 30% of the trajectory frames. Non-catalytic residues are highlighted in italics.

Donor	Acceptor	Occurrence, %
Glu–124/N–H	*Val*–166/O	90
Tyr–63/O4–H	Glu–233/O$_{\delta}$	56
Arg–128/N–H	Glu–124/O	89
Ala–191/N–H	Glu–233/O	99
Asn–190/N$_{\gamma}$–H	Glu–124/O$_{\delta'}$	37
Asn–190/N$_{\gamma}$–H	Glu–124/O$_{\delta}$	36

Supplemental data S8.

Effect of C310A substitution on the structure and dynamics of Bgl2p

C310A substitution lead to the disappearance of the disulfide bond with C262, that increases the conformational mobility of the C-terminal region 306-313 (Figure S7.)

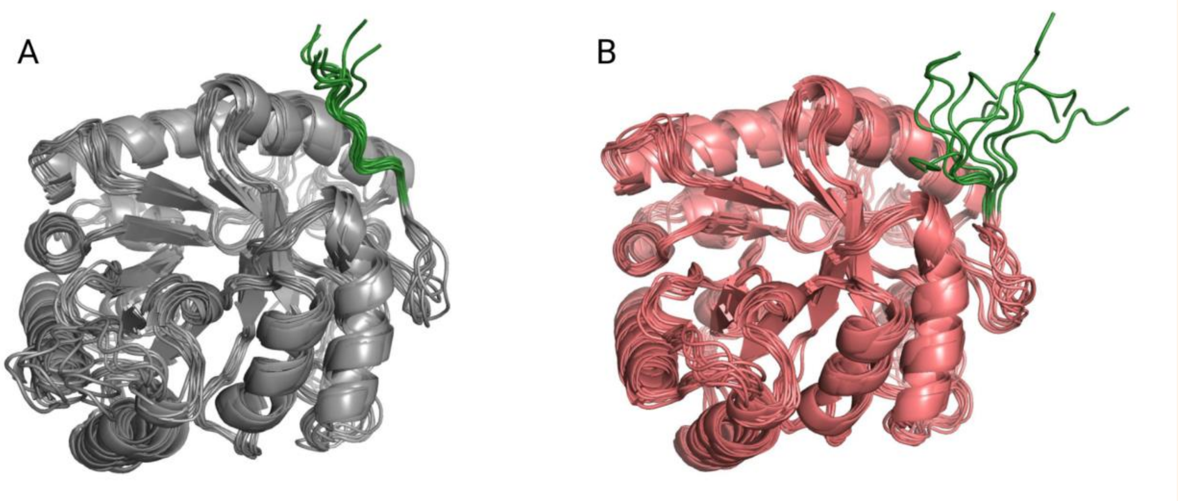

Figure S7. The conformational mobility of C-terminus 306-313 (marked in green) of Bgl2p structures, non-mutated (A) and with C310A substitution (B). Structural alignment for Bgl2p structures for frames from molecular dynamics trajectory.
