# Supplementary figures and images for: Yeast Glucan Remodeling Protein Bgl2p: Amyloid Properties and the Mode of Attachment in Cell Wall
Source: Int J Mol Sci. 2024 Dec 22;25(24):13703. doi: 10.3390/ijms252413703 (PMC11677059; doi:10.3390/ijms252413703)

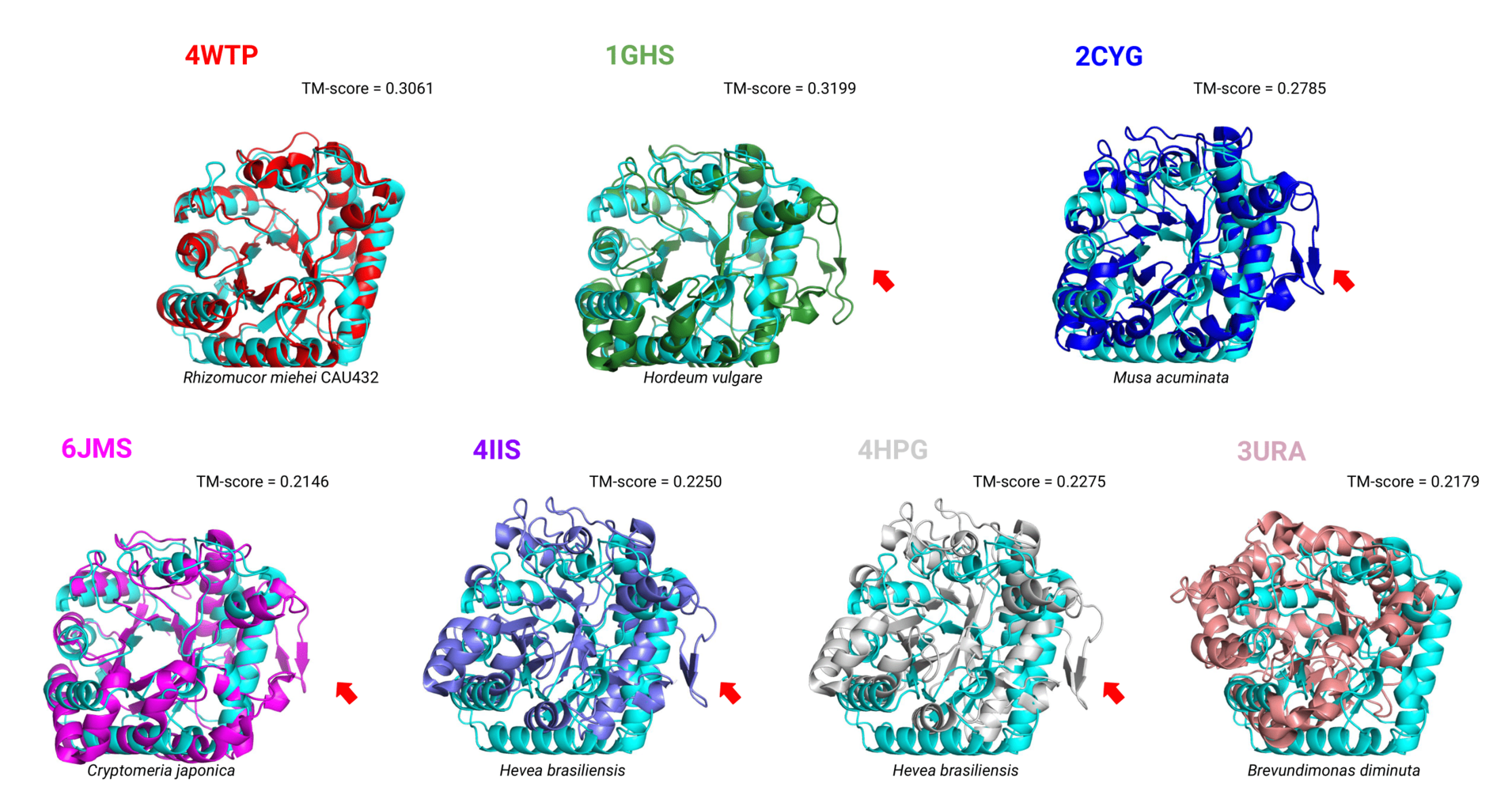

Supplement: Supplementary file 1 [file ijms-25-13703-s001.zip › Figure S1.tiff]

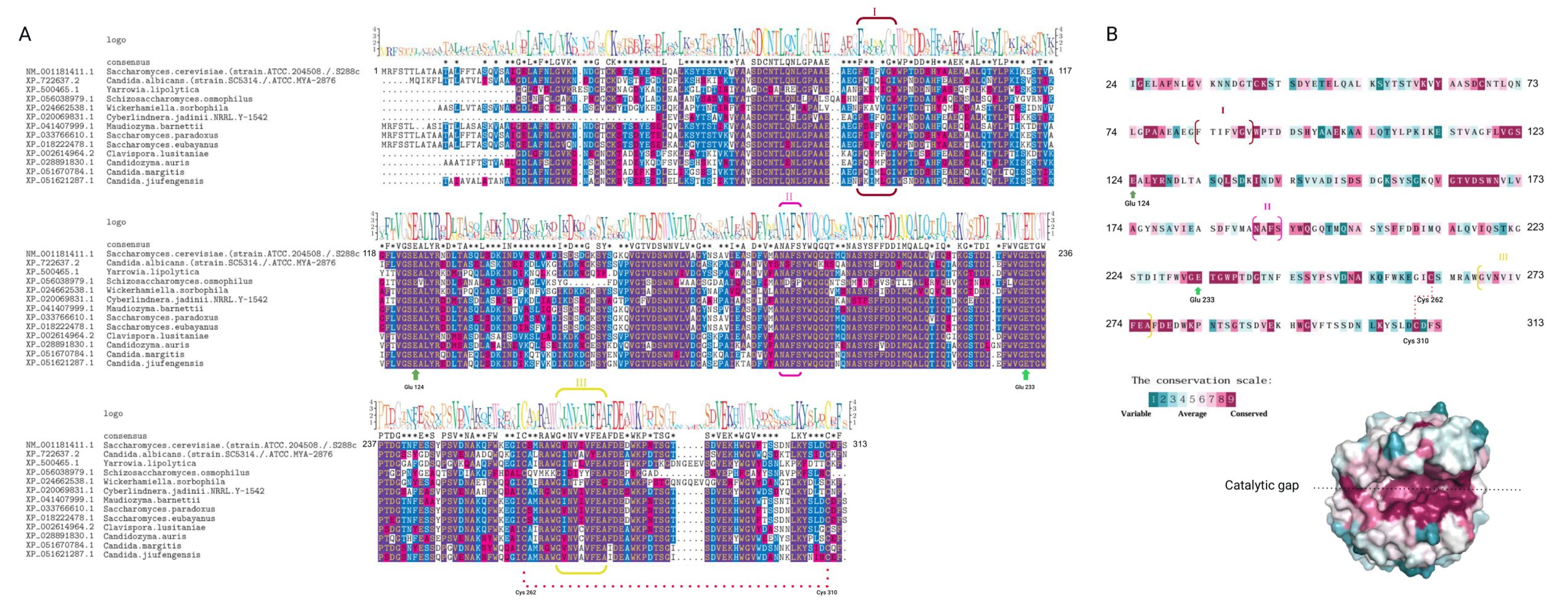

Supplement: Supplementary file 1 [file ijms-25-13703-s001.zip › Figure S2.tiff]

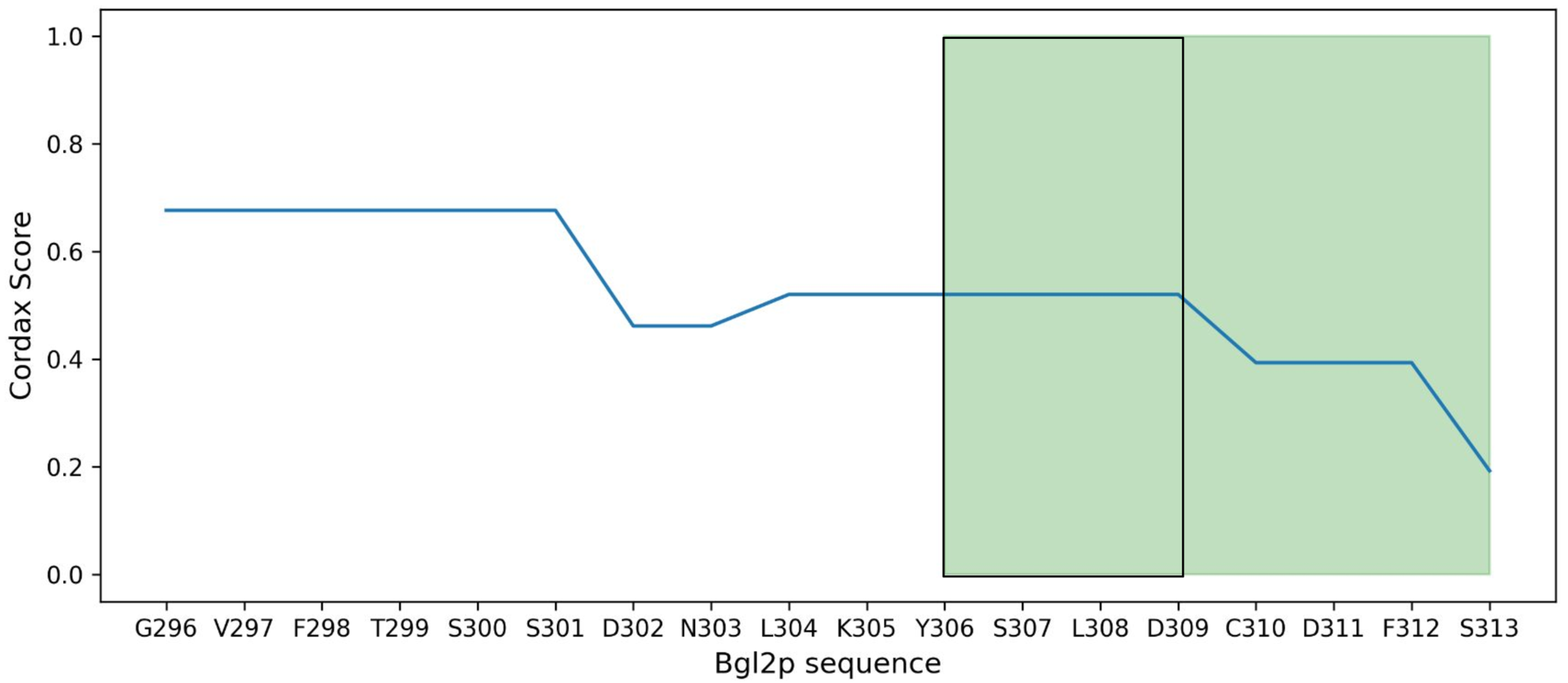

Supplement: Supplementary file 1 [file ijms-25-13703-s001.zip › Figure S3.tiff]

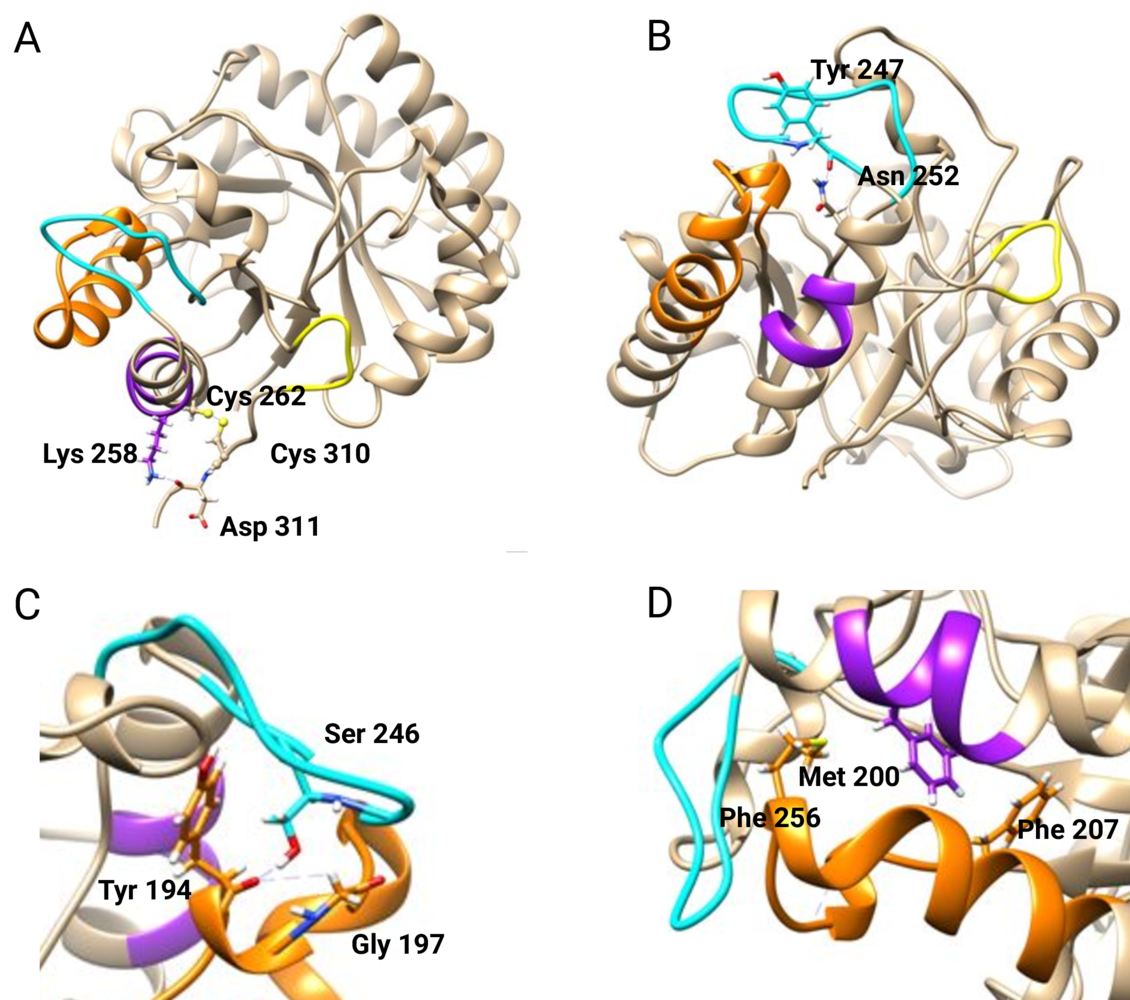

Supplement: Supplementary file 1 [file ijms-25-13703-s001.zip › Figure S4.tiff]

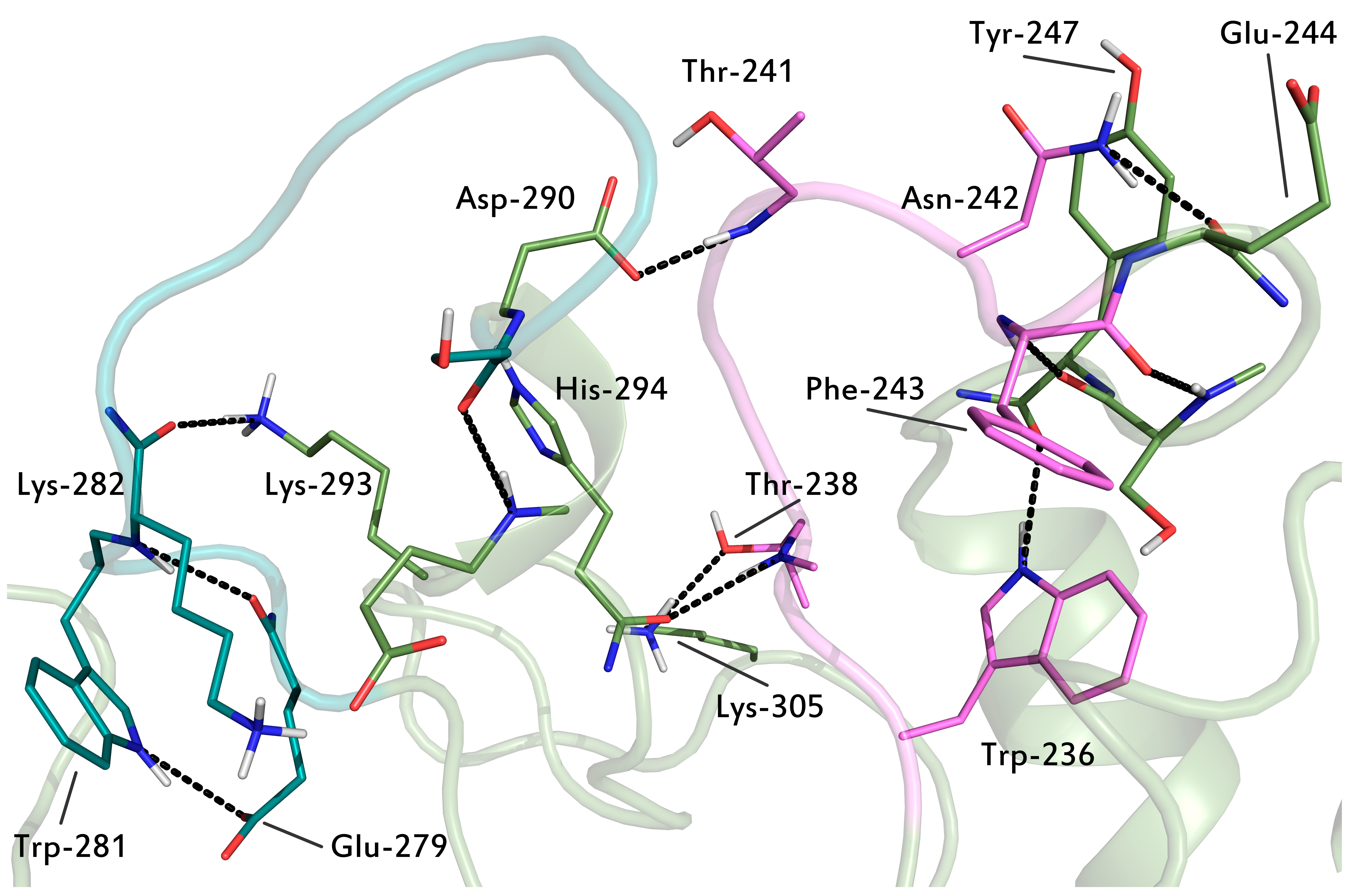

Supplement: Supplementary file 1 [file ijms-25-13703-s001.zip › Figure S5.tif]

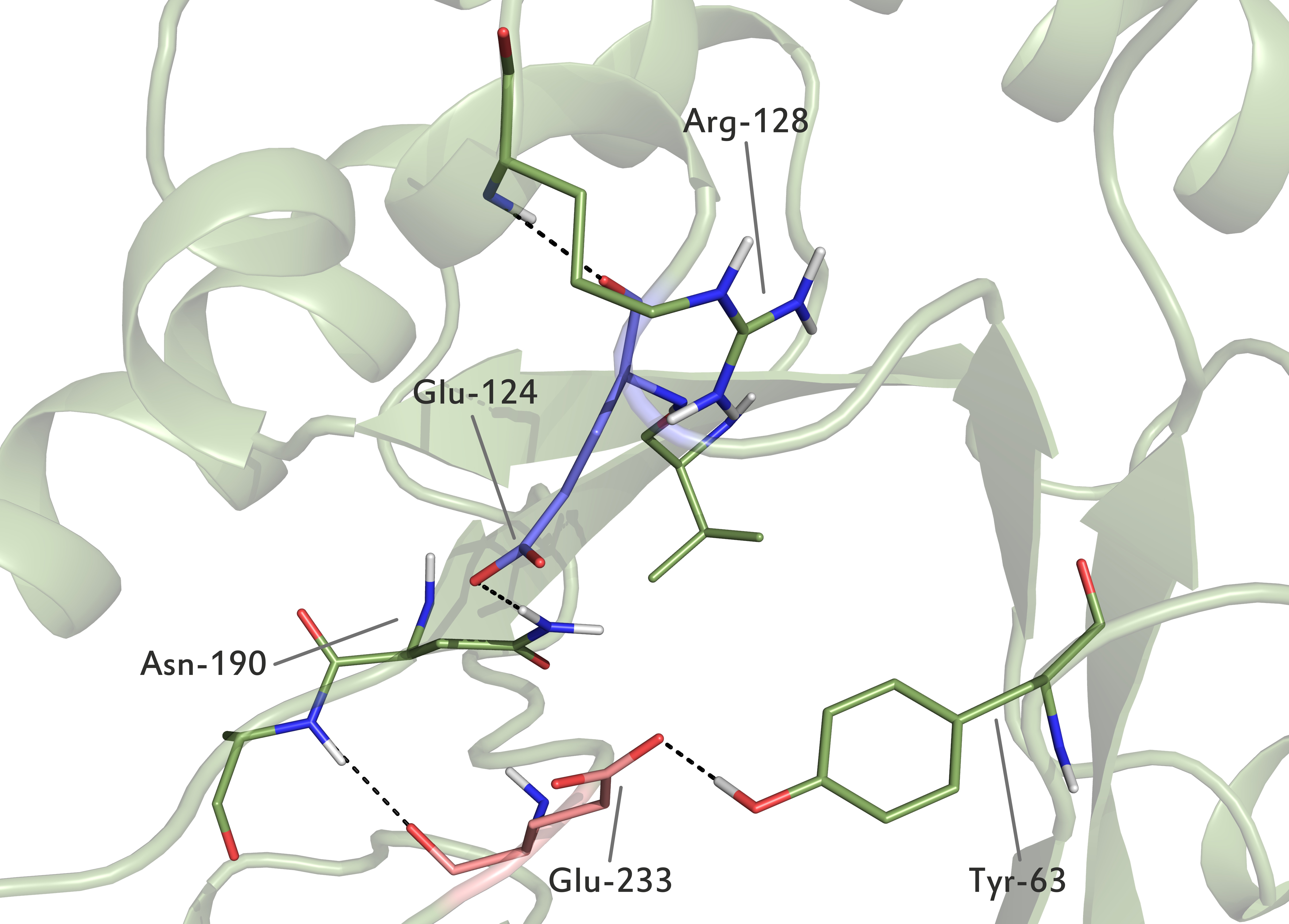

Supplement: Supplementary file 1 [file ijms-25-13703-s001.zip › Figure S6.tif]

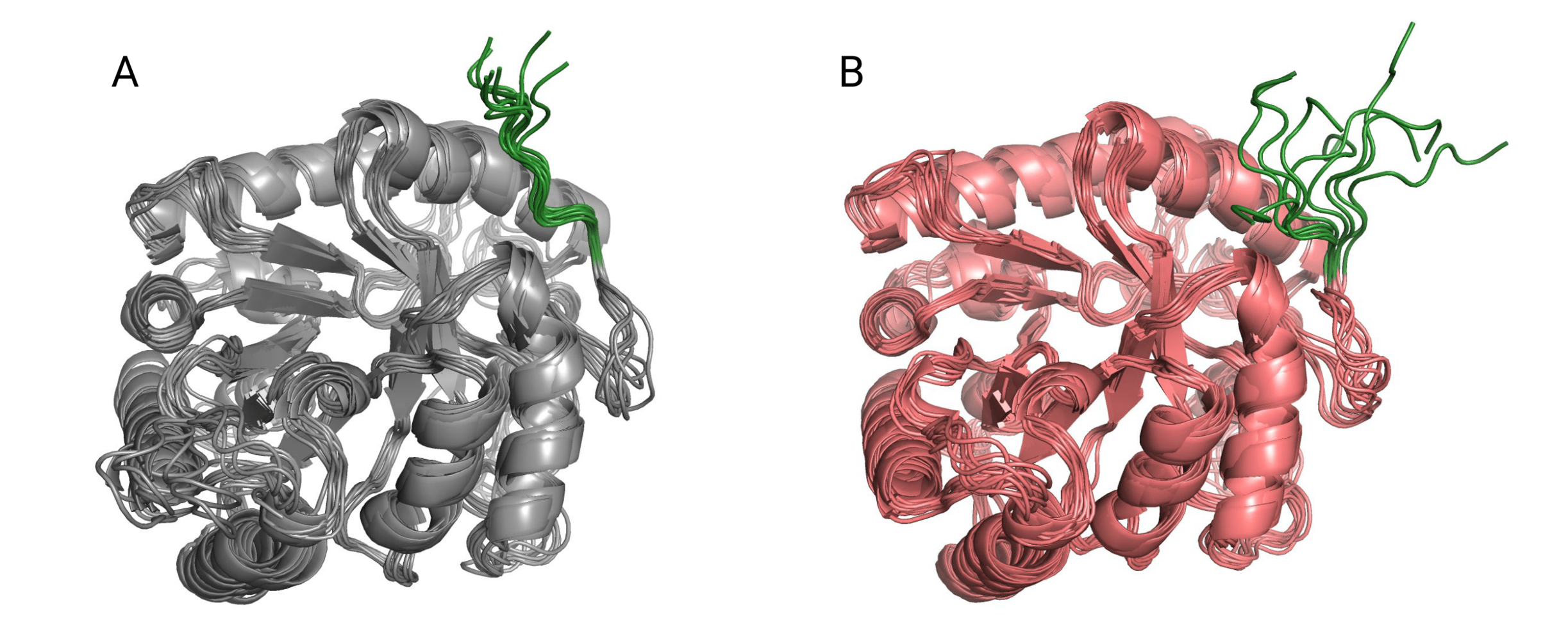

Supplement: Supplementary file 1 [file ijms-25-13703-s001.zip › Figure S7.tiff]
